# Supplementary material for: Synaptic counts approximate synaptic contact area in Drosophila
Source: PLoS One. 2022 Apr 4;17(4):e0266064. doi: 10.1371/journal.pone.0266064 (PMC8979427; doi:10.1371/journal.pone.0266064)
Supplement: S1 File — A compressed archive containing the Bayesian analysis code as an R package. (ZIP) [file pone.0266064.s001.zip › Rpackage/Readme.pdf]

D. Bonnéry

1 Model and notations

We observe for a series of $n = 80$ neurons pairs $(i < j)$, the number of contacts $n_{(i,j)}$ and $Y_{(i,j)} = \sum_{k=1}^{n_{(i,j)}} Y_{i,j,k}$, where $Y_{i,j,k}$ is the synaptic area for contact k from i to j. The goal here is to study the use of $n_{(i,j)}$ to predict the synaptic area $Y_{(i,j)}$. Counting the contacts is less expensive than measuring the synaptic area and one wants to know if number of contacts is a good proxy.

Assume a latent model $Y_{i,j,k} \sim \mathcal{N}(\beta, \sigma^2)$ are i.i.d, $\beta \in \mathbb{R}^+$, implies that the latent model for the distribution of $Y_{i,j} \mid n_{i,j}$ is $Y_{i,j} \mid n_{i,j} \sim \mathcal{N}(\beta n_{i,j}, n_{i,j}\sigma^2)$ This is a very standard model and the parameters can be estimated with the Weighted Least Square (WLS) method, that gives the Best Linear Unbiased Estimators for this model.

We obtain a coefficient of determination $R^2 = 0.903$ with three significant digits.

Figure 1: Residual analysis

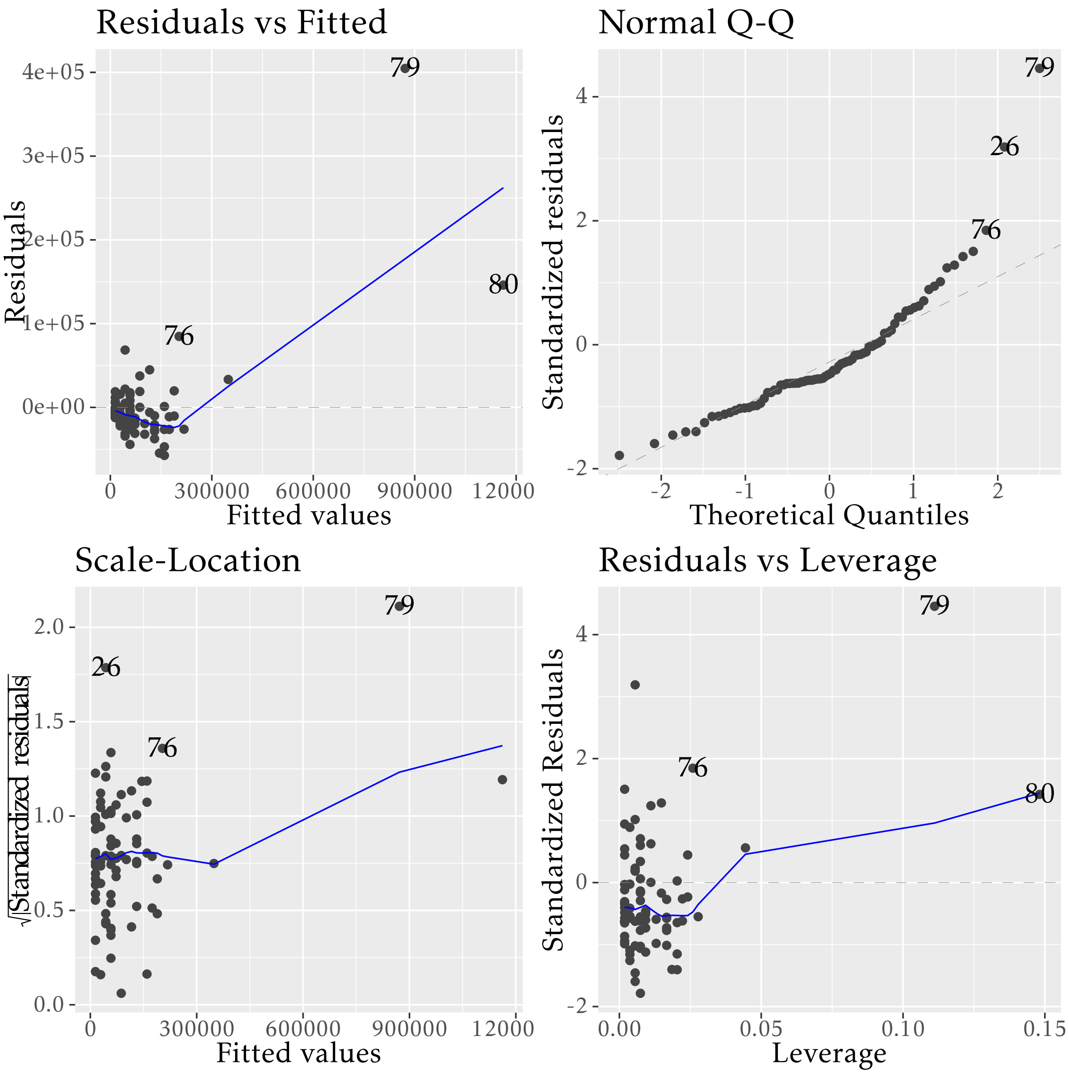

The visual analysis of residuals in Figure 1 provides strong evidence that: The normality approximation seems very reasonable (see QQ plot), There does not seem to be a correlation between the standardized residuals and the fitted values, which means that our assumption on the variance on the residuals conditional to the covariates is very reasonable.

We reproduced the study after eliminating identified leverage points. We obtain a coefficient of determination $R^2 = 0.905$ with three significant digits). The value for a new estimate of β is $\hat{\beta}^{(-\text{leverage})} = 14700$ with 3 significant digits, to compare with $\hat{\beta} = 14500$ (three significant digits), which makes a relative difference of -1.01%.

Figure 2: Residual analysis

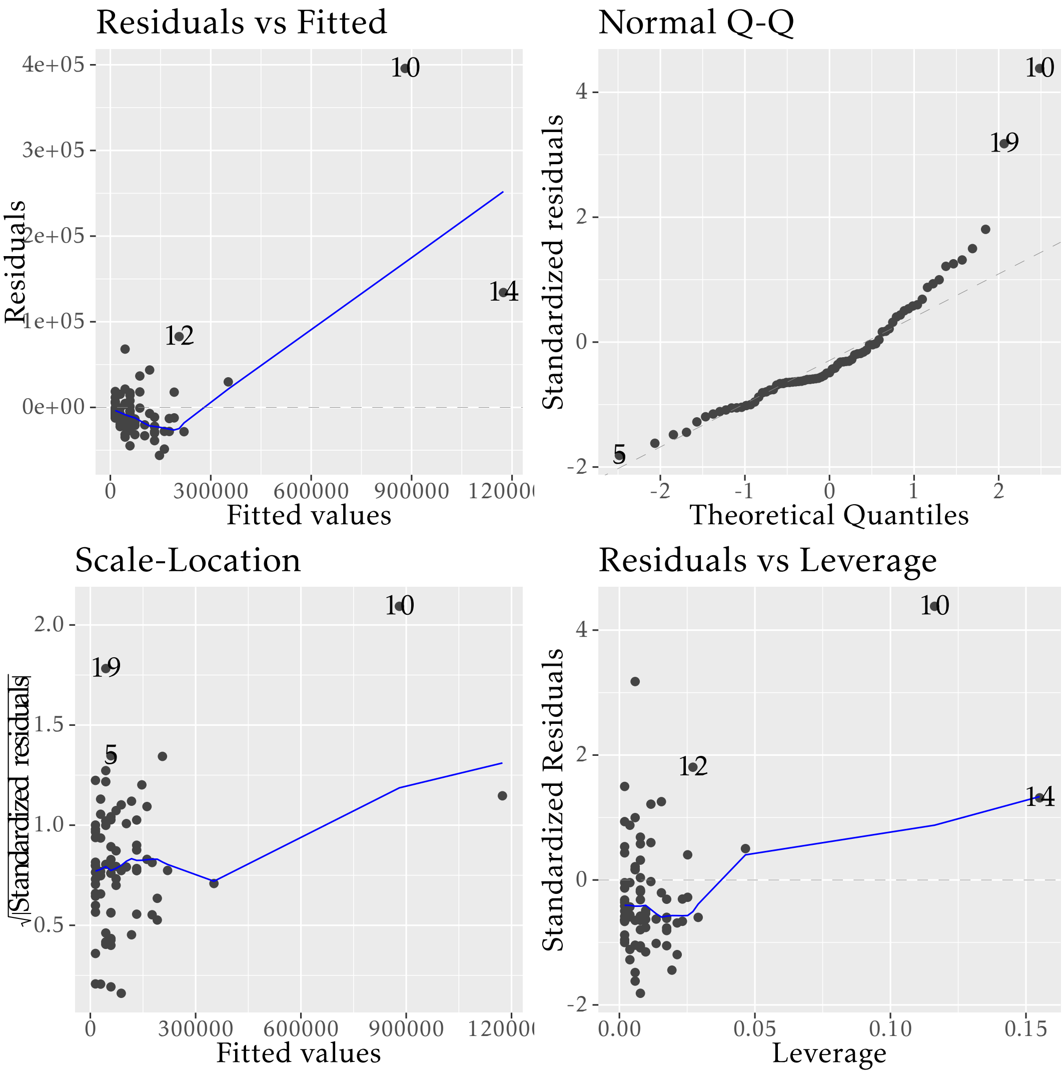

2 Estimating prediction error

The Weighted Least Square estimation procedure gives us an estimate $\hat{\sigma} = 12400$ with three significant digits.

The expeted value of the squared prediction error of the area for a new value x of counts is $\mathrm{E}\left((x(\hat{\beta} - \beta) + \varepsilon)^2 \mid X = x\right) = x^2 \mathrm{Var}\left(\hat{\beta}\right) + x\sigma^2$. It is estimated by $x^2 \hat{\sigma}_{\hat{\beta}}^2 + x\hat{\sigma}^2$.

The estimated prediction confidence interval for a new value of the number of contacts x is given by the formula:

$$\mathrm{PI}_{1-\alpha}(Y \mid X = x) = \left[x\,\hat{\beta} \mp q_{t,n-1}(1-\alpha/2)\sqrt{x\,\hat{\sigma}^2 + x^2\,\hat{}^2_{\hat{\beta}}} \right],$$

where $q_{t,n-1}(1-\alpha/2)$ is the $1-\alpha/2$ quantile of the for a student distribution with n parameters, and $\hat{}_{\hat{\beta}} = 535$, with three significant digits, is given by the Weighted Least Square Error procedure.

Figure 3 represents the 95% level prediction intervals for different values of x.

Figure 3: Residual analysis

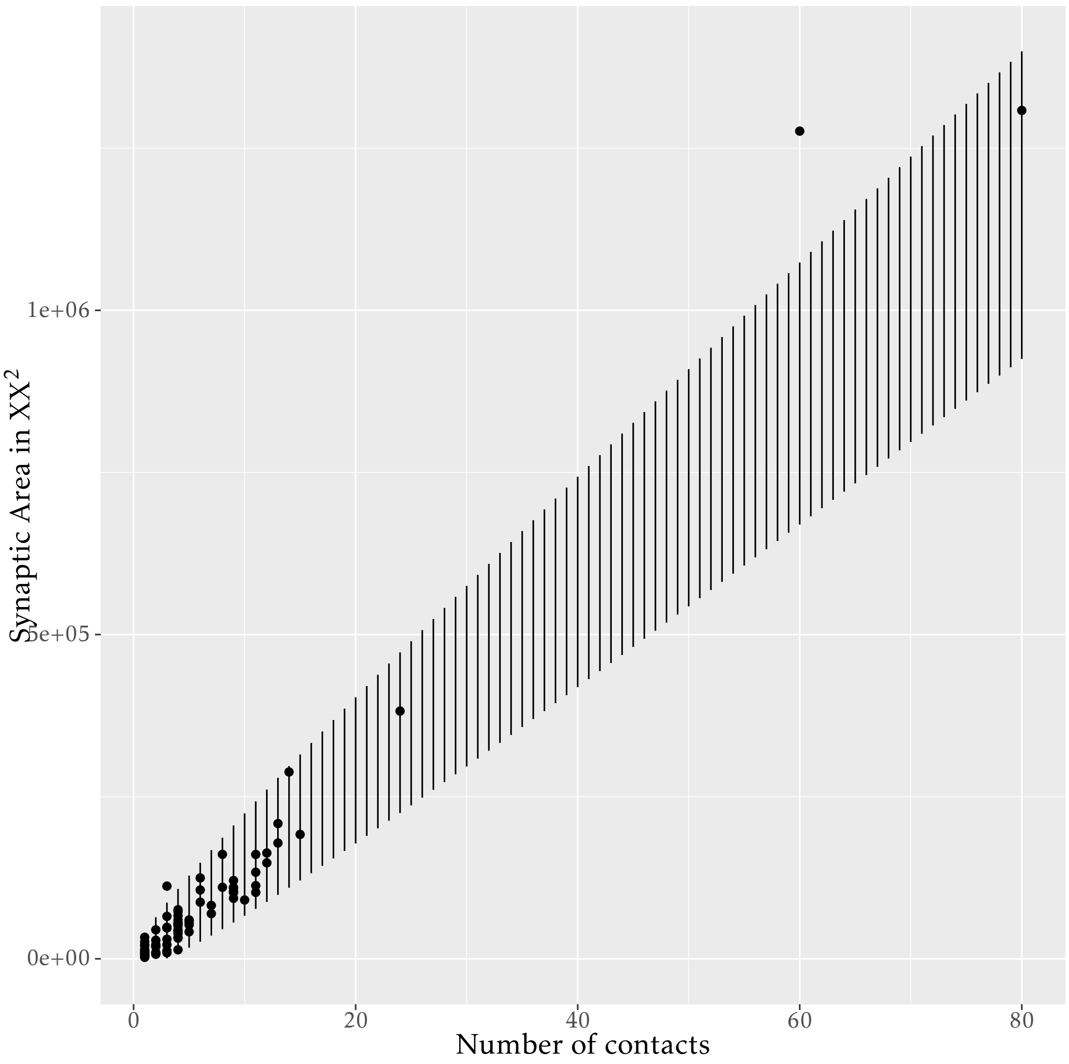

Figure 4 and 5 represents the prediction interval lower and upper boundaries for $\alpha \in \{.9, .95, .99, .999\}$.

Figure 4: Residual analysis

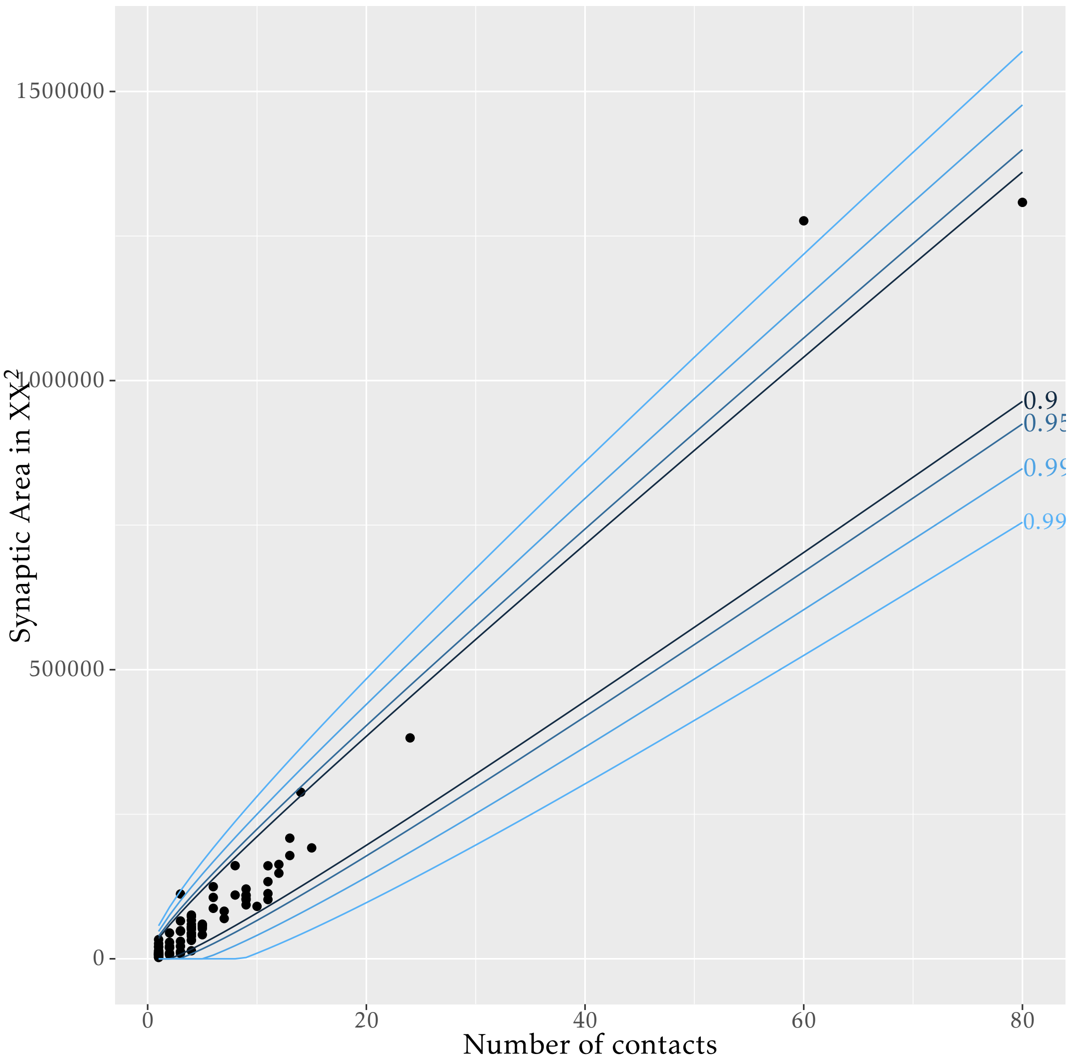

Figure 5: Residual analysis

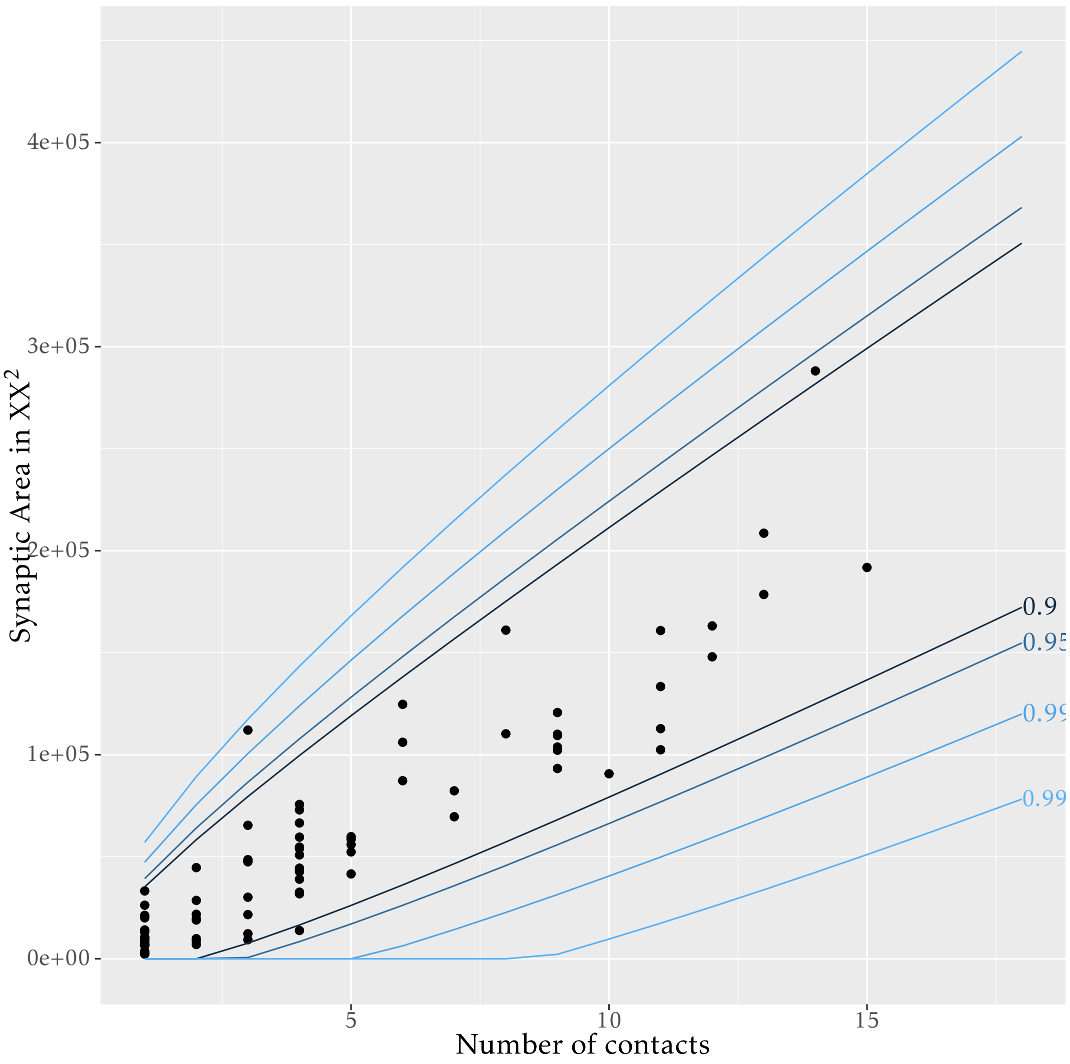

3 Refinements

3.1 Hierachical model

The goal of the study is just to measure how good a proxy for the total surface is the number of connections. The prediction intervals obtained from a simple linear model above are an accurate way to measure this. Nevertheless, one may want to obtain more refined prediction intervals. The different leverages for this goal are the following: 1. Using the auxiliary information brought by the type of neuron, 2. Modeling the surface with a Gamma distribution, as the areas are positive variables.), 3. Use all the information at the contact level, not the aggregated information at the pair level (for a Gamma distribution, the sufficient information is the sum over the sample and the sum over the log-transformed sample.

For this goal we use the following hierarchical model:

Level 1. $Y_{i,j,k} \mid n_{i,j}, X_{i,j}, \text{scale}_{0,(i,j)}, \text{rate}_{0,(i,j)} \sim \Gamma_{\text{scale}_{0,(i,j)},\text{shape}_0}$ and conditionnally on $\text{scale}_{0,.}, \text{shape}_0$ the $Y_{i,j,k}$ are independent accross i, j, k. It means that for each pair, the distribution of the areas for each contact point follows the same distribution. This allows to take into account that between two pairs, the distribution of surfaces can be the output of different distributions.

Level 2. $\text{scale}_{0,(i,j)} \mid X_{(i,j)} = x \sim \Gamma_{\text{scale}_{1,x},\text{shape}_1}$:conditionnally on $\text{scale}_{1,.}$ and rate_1, the scale parameters for the first level (the pair level) are distributed according to a Gamma distribution, whose parameters depend on the circuit only. This allows to say that the parameters for each pair are the output of a random process, and that a different process is at work for different types of neurons. Doing so, the fitted model is more likely to apply to another kind of circuit that was not in the data when the model was fitted.

Level 3. $\text{scale}_{1,x} \sim \Gamma_{\text{scale}_2,\text{shape}_2}$: For each circuit, the scale parameters of the distribution of the pair of parameters follow a gamma di$P^{Y|i,j,X_{i,j},n_{i,j}} = P^{Y|X,A}$.

Level 4. We use a flat prior for all the hyperparameters scale_2, rate_0, rate_1, rate_2.

By using this model, we will take into account the variability accross circuits and the variability accross pairs. The total variability can be decomposed between the extra circuit, intra-circuit, intra pair variability. By fitting the simple model $Y \sim \Gamma_{sc,sh}$ on the whole population, the variability that we will measure will be an average of the total variability, but for a certain selection of circuits and pairs only. This simple model would fit the data it has been fitted on better than the hierarchical model. But one should trust the hierarchical model better for a pair of neuron that was not present in the training dataset.

3.2 results

In this section, we plot the 95% confidence prediction intervals for the total connection surface given a number x of connections in "pm^2 ?" knowing the circuit, or not knowing the circuit.

Figure 3.2 and 3.2 are the confidence intervals obtained via MCMC derived from the hierarchical model.

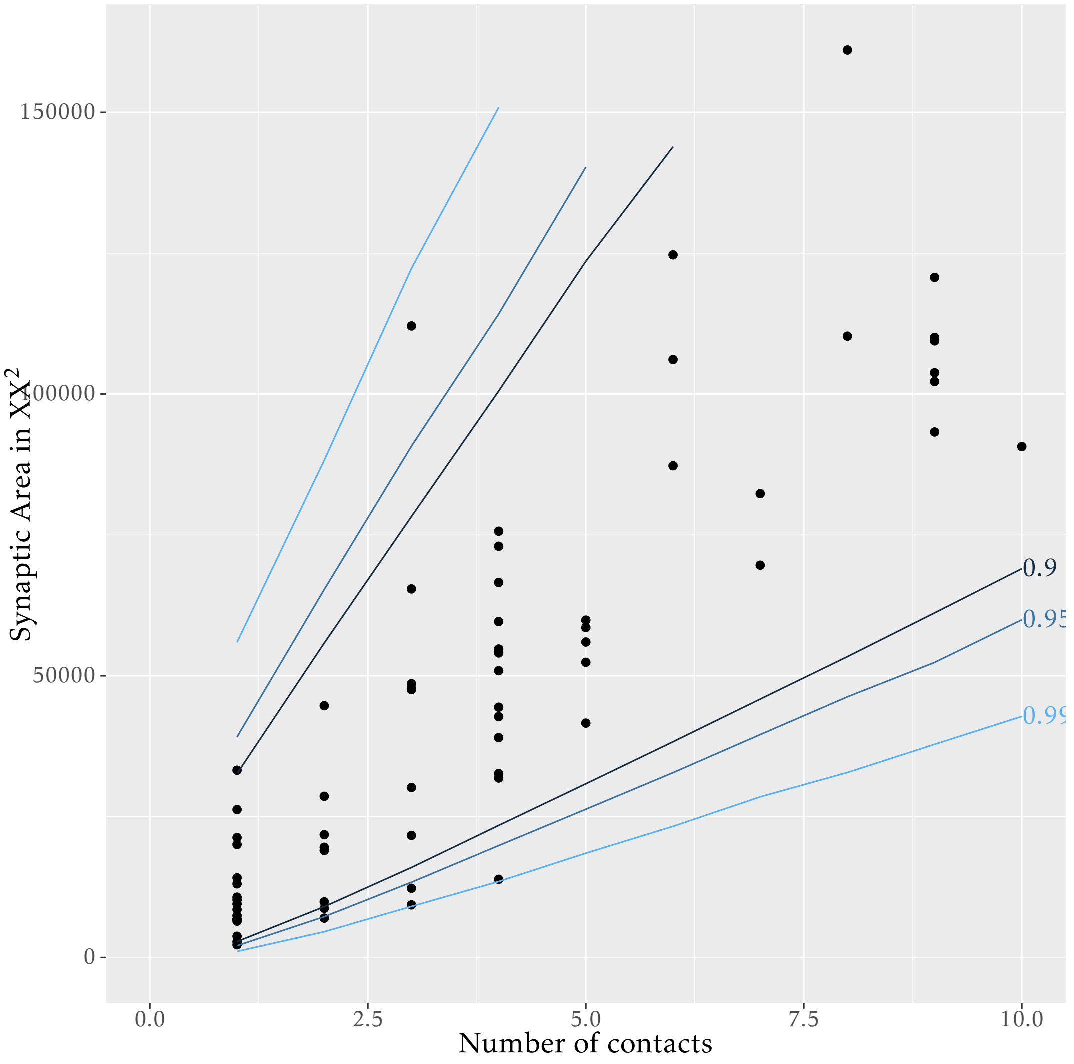

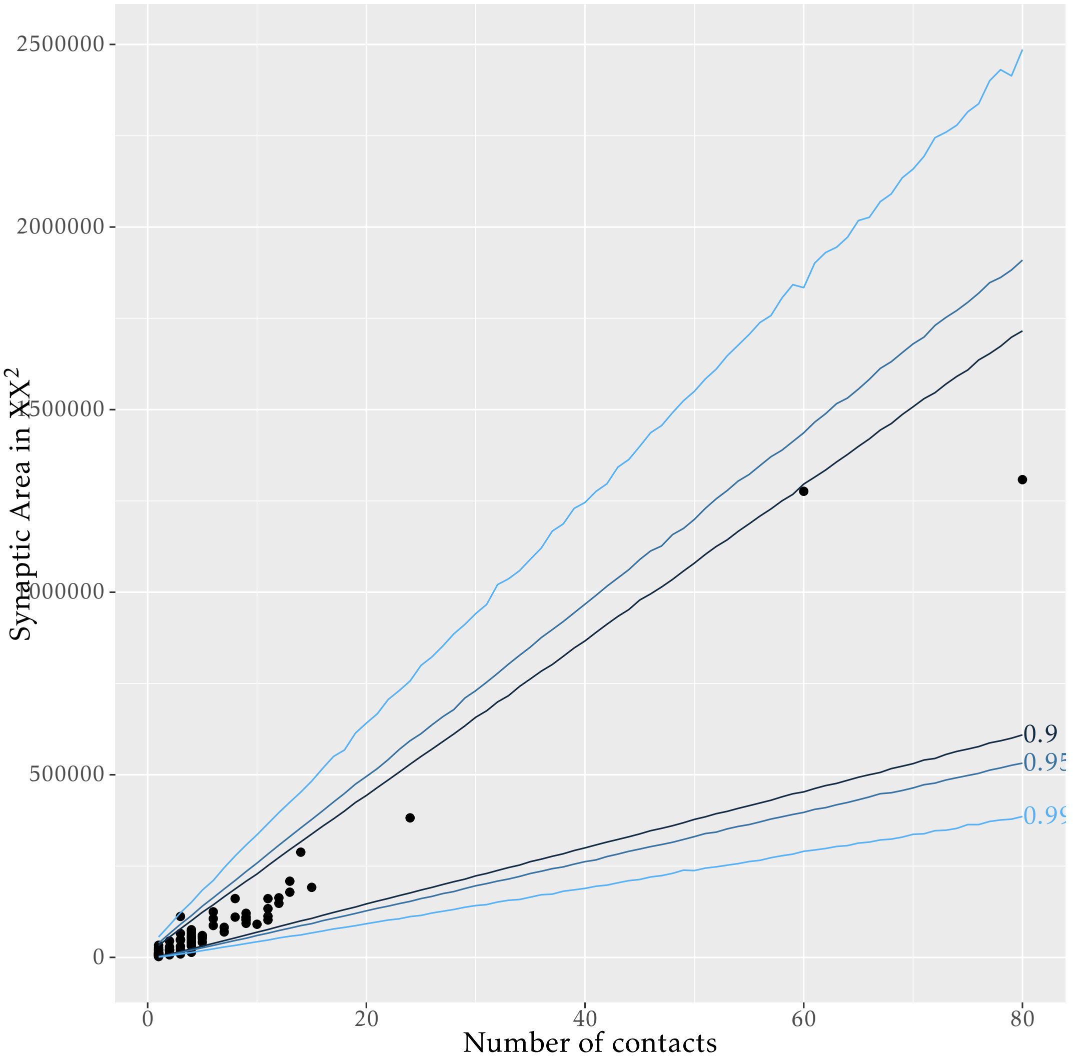

2500000
2000000
1500000
1000000
500000
0
0
20
40
60
80
Number of contacts
Synaptic Area in XX²
0.9
0.95
0.99

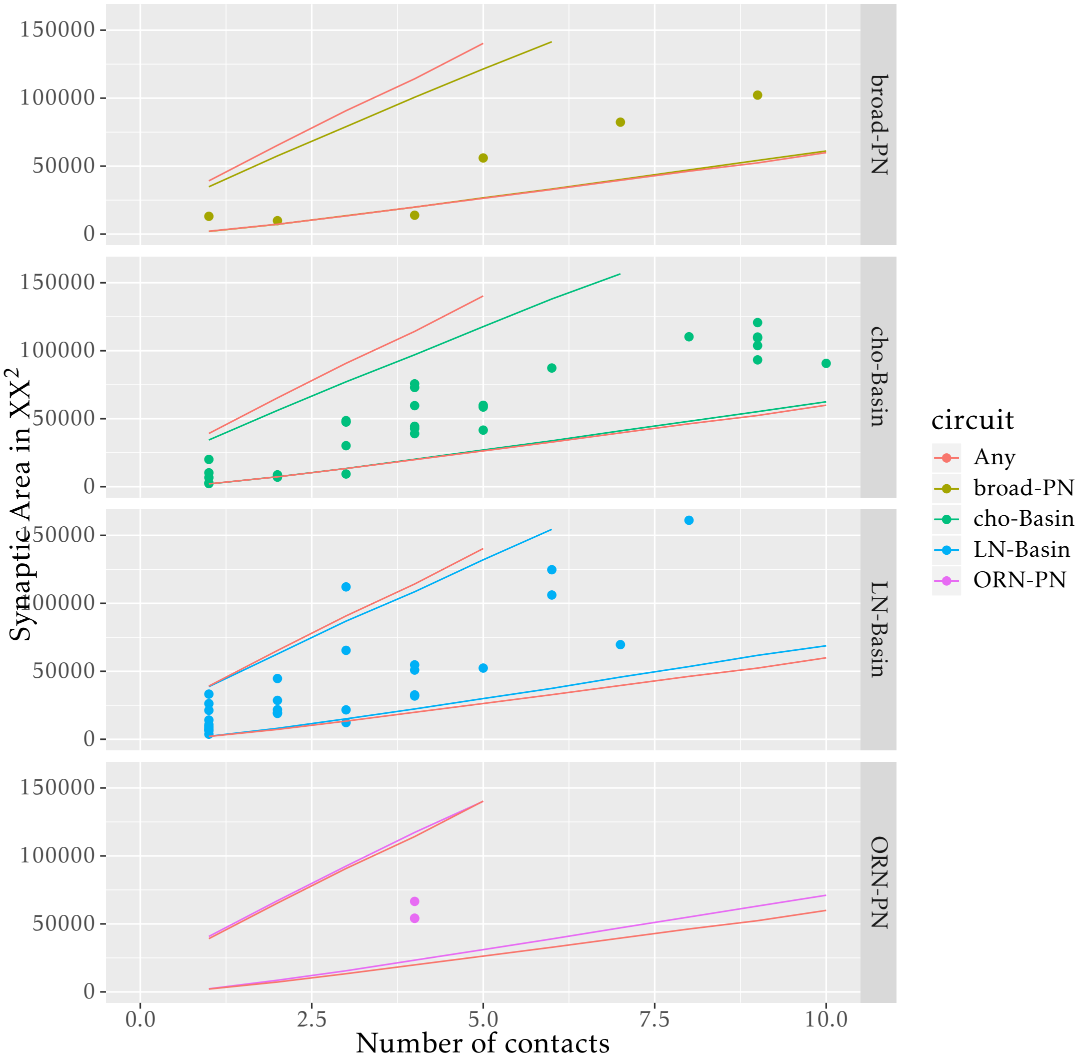

4 Same thing but frequentist approach, simpler.

4.1 Model

If we do not want to go for Bayesian, we can use a frequentist approach with a mixed model on $\ln(Y)$.

$$\ln(Y_{i,j,k}) = a + b_{i,j} + c_{X_{i,j}} + \varepsilon_k$$

where $b_{i,j}$ and $c_{X_{i,j}}$ are random effects. $b_{i,j} \sim \text{Normal}(0, \sigma_1^2)$, $c_x \sim \text{Normal}(0, \sigma_2^2)$, and $\varepsilon_k \sim \text{Normal}(0, \sigma_0^2)$.

The mixed model fitting will provide estimates for a, σ_0^2, σ_1^2 and σ_2^2, as well as an estimator of the variance of the estimator of a_0, $\hat{\sigma}_a^2$.

The prediction interval at level (α) of $\ln(Y)$ correspond to the inter quantiles interval for the $(1-\alpha)/2$ and $1-(1-\alpha)/2$ quantiles of a lognormal distribution of parameters $\mu = a$, $\sigma^2 = \hat{\sigma}_a^2 + \hat{\sigma}_0^2 + \hat{\sigma}_1^2 + \hat{\sigma}_2^2$.

The sum of x lognormal i.i.d. lognormal random variables is difficult to approximate for x small, but one can compute the confidence interval via Monte Carlo.

Figure 4.1 and 4.1 represent the prediction intervals for different confidence levels.

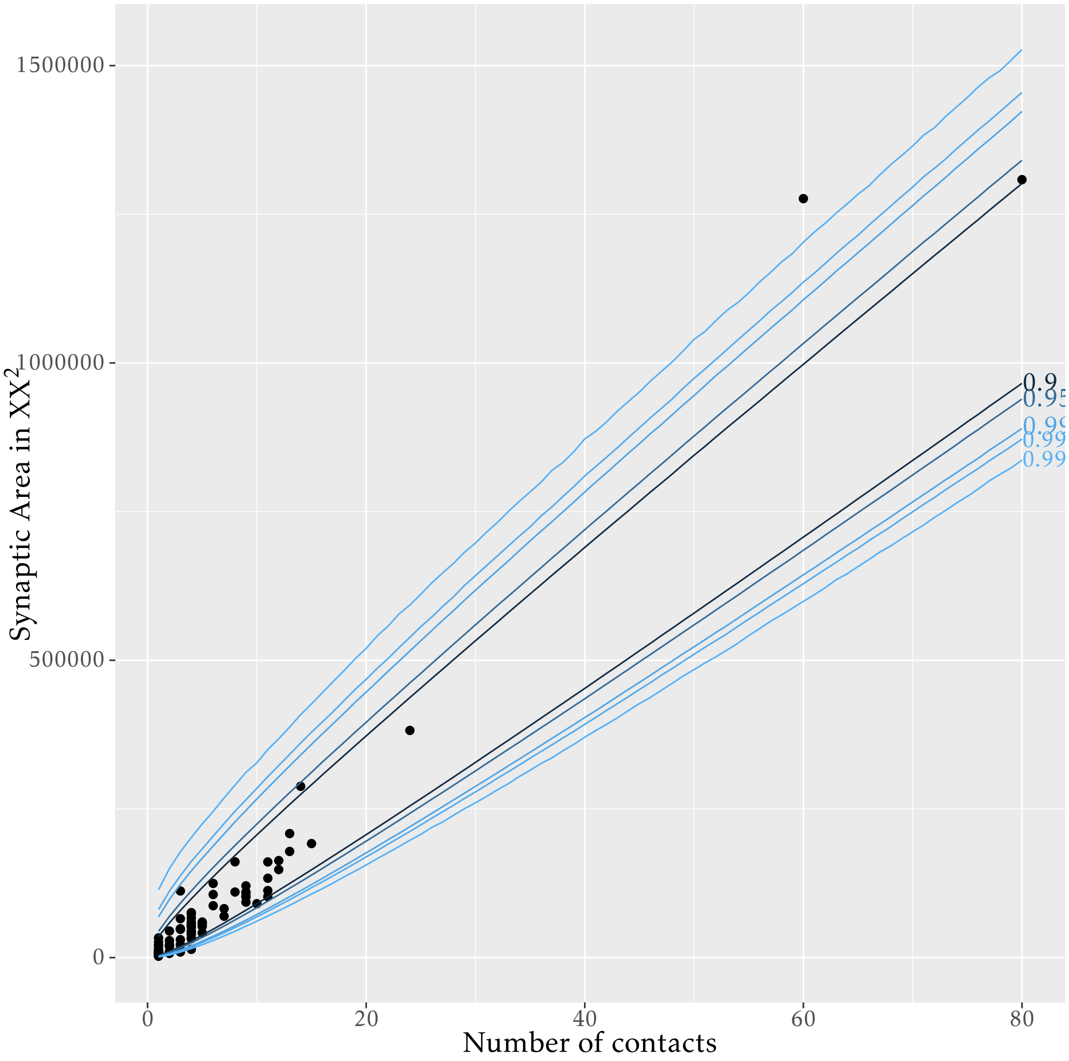
1500000
1000000
500000
0
0
20
40
60
80
Number of contacts
Synaptic Area in XX^2
0.9
0.95

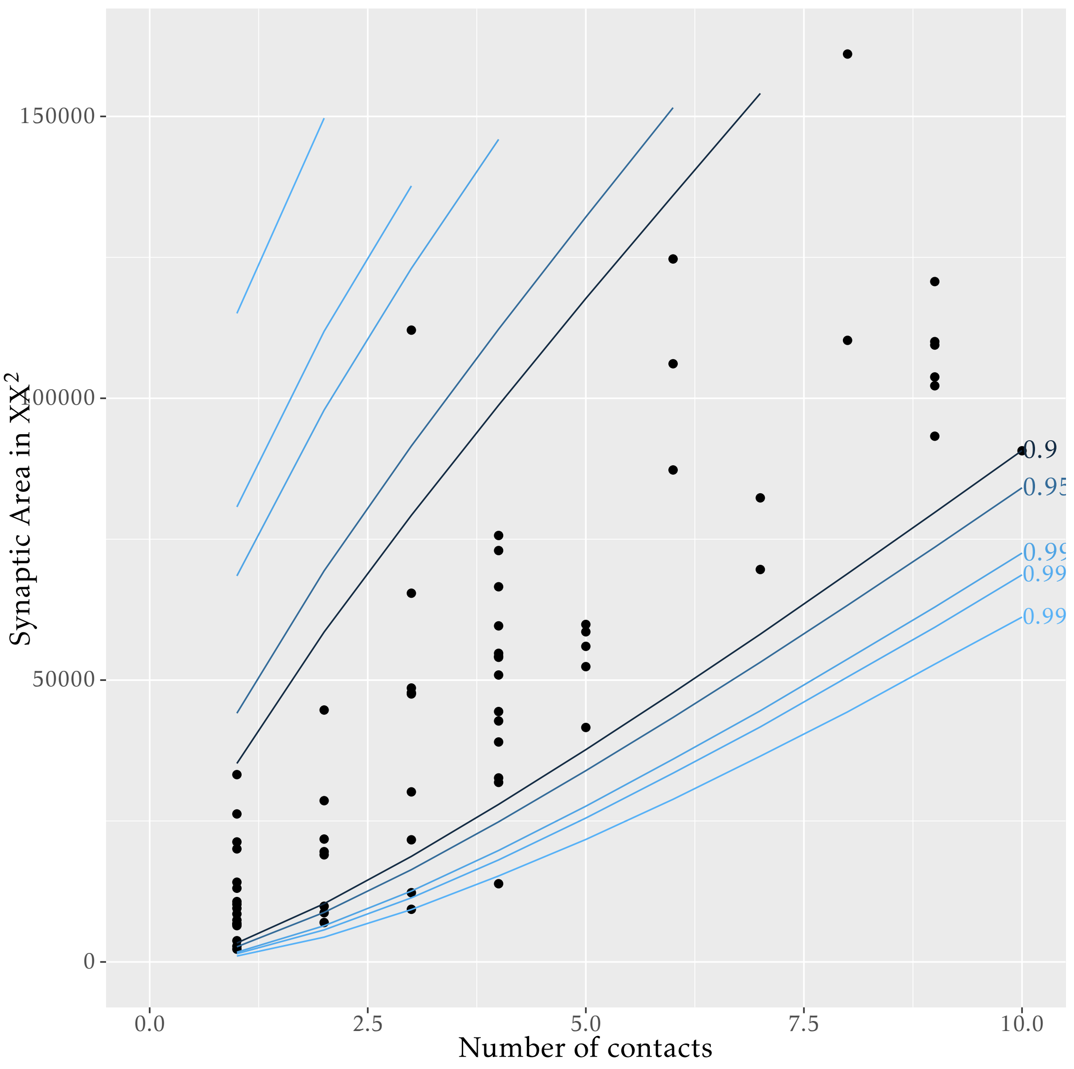

Conclusion

We conclude that the prediction confidence intervals obtained with a simple linear model give an accurate way of measuring the incertitude with respect to the predition of a contact area by the number of synapses. If more precision is needed, the hierarchical models we propose fit the data better, and the prediction intervals obtained also apply to neurons in circuits that were not present in the training dataset, provided the assumption that the random effects associated to the circuit come from the distribution specified in each model. Another advantage of hierarchical modeling is that it allows to propose more accurate prediction intervals for circuits that were present in the training dataset.

5 Rcode

```
demo("NormalModel",package="synapsearea")
demo("BayesianHierarchicalGamma",package="synapsearea")
demo("LognormalModel",package="synapsearea")
```

6 More graphs

6.1 Graphs from linear model

Figure 6:

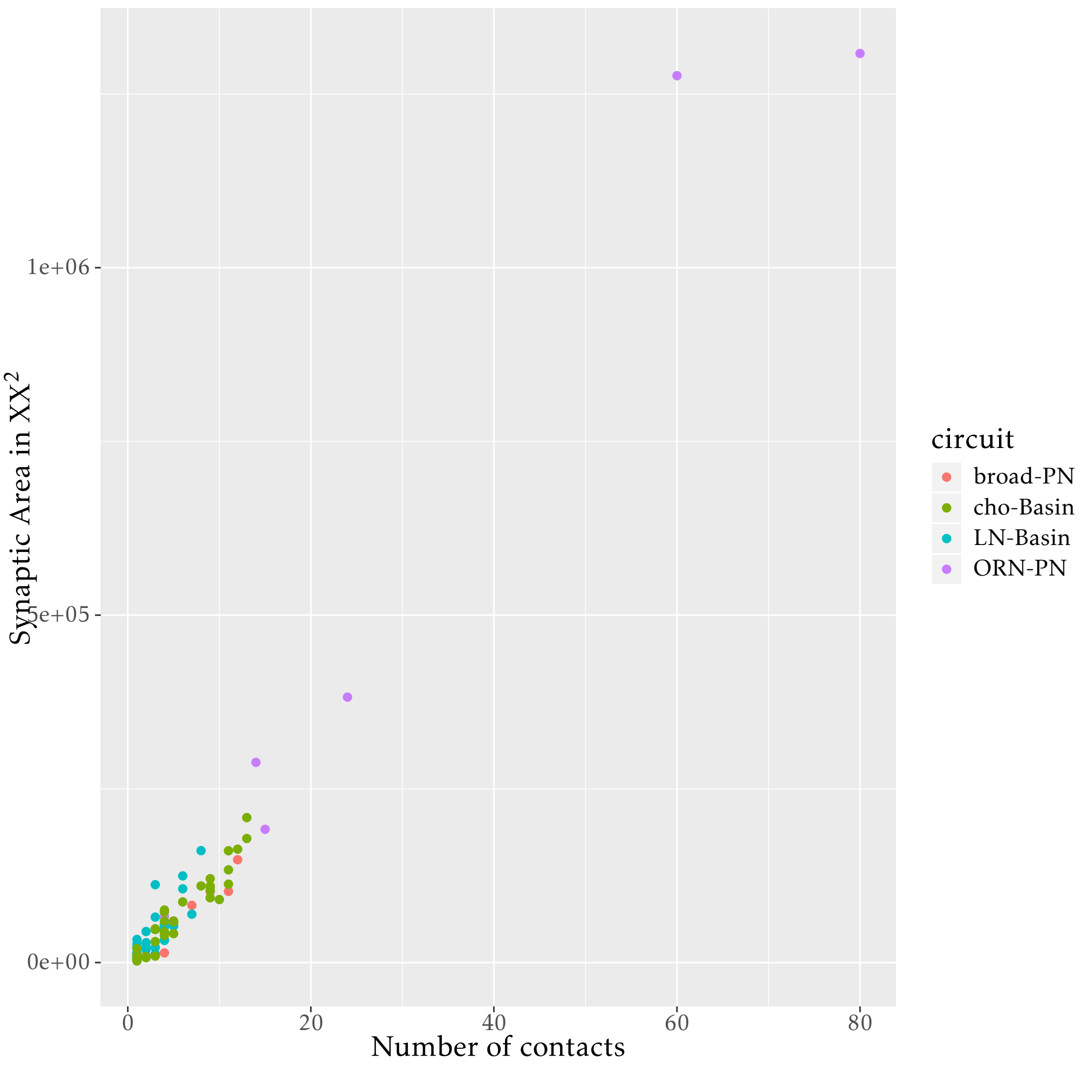

Figure 7:

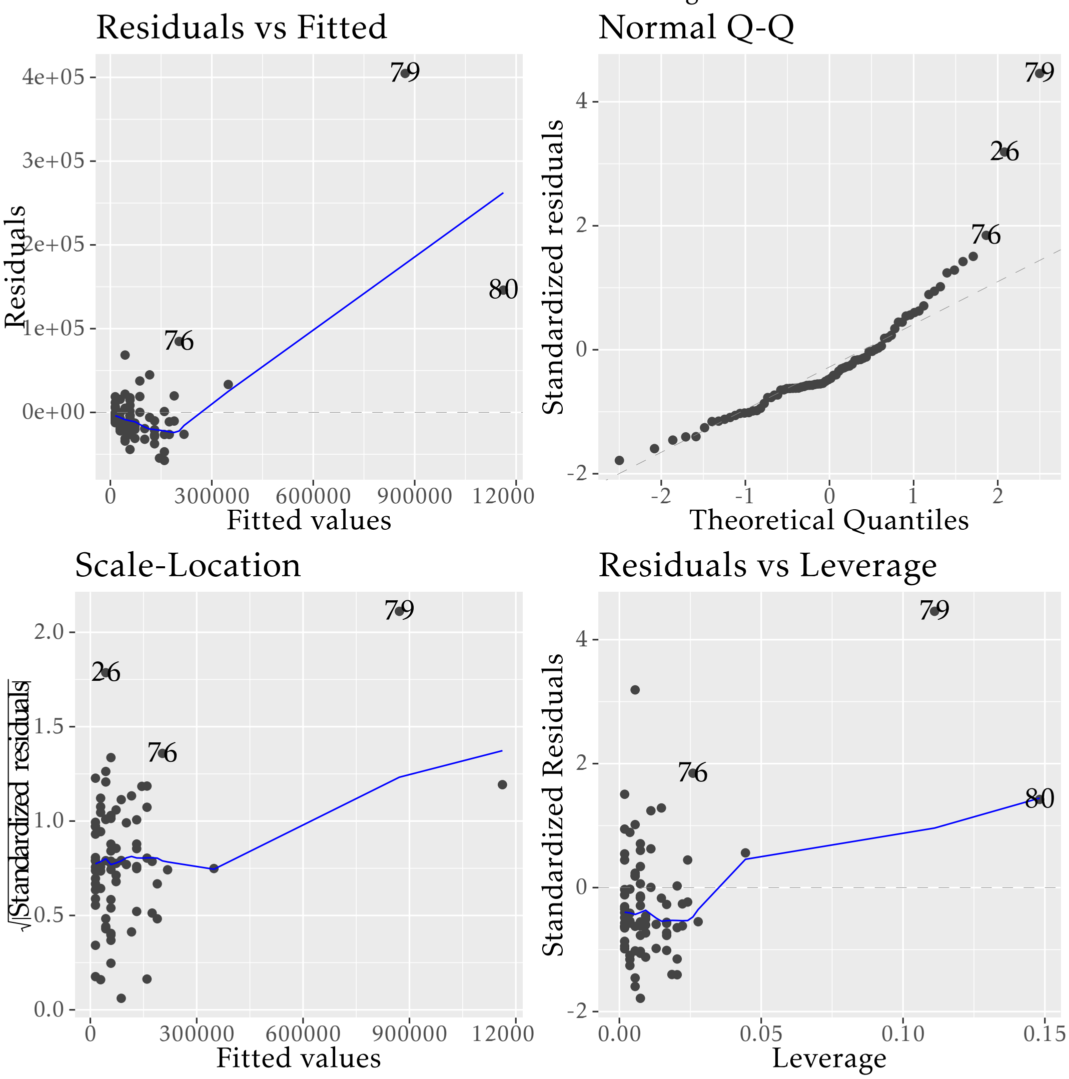
Residuals vs Fitted
Residuals
Fitted values
Normal Q-Q
Standardized residuals
Theoretical Quantiles
Scale-Location
√|Standardized residuals|
Fitted values
Residuals vs Leverage
Standardized Residuals
Leverage

Figure 8:

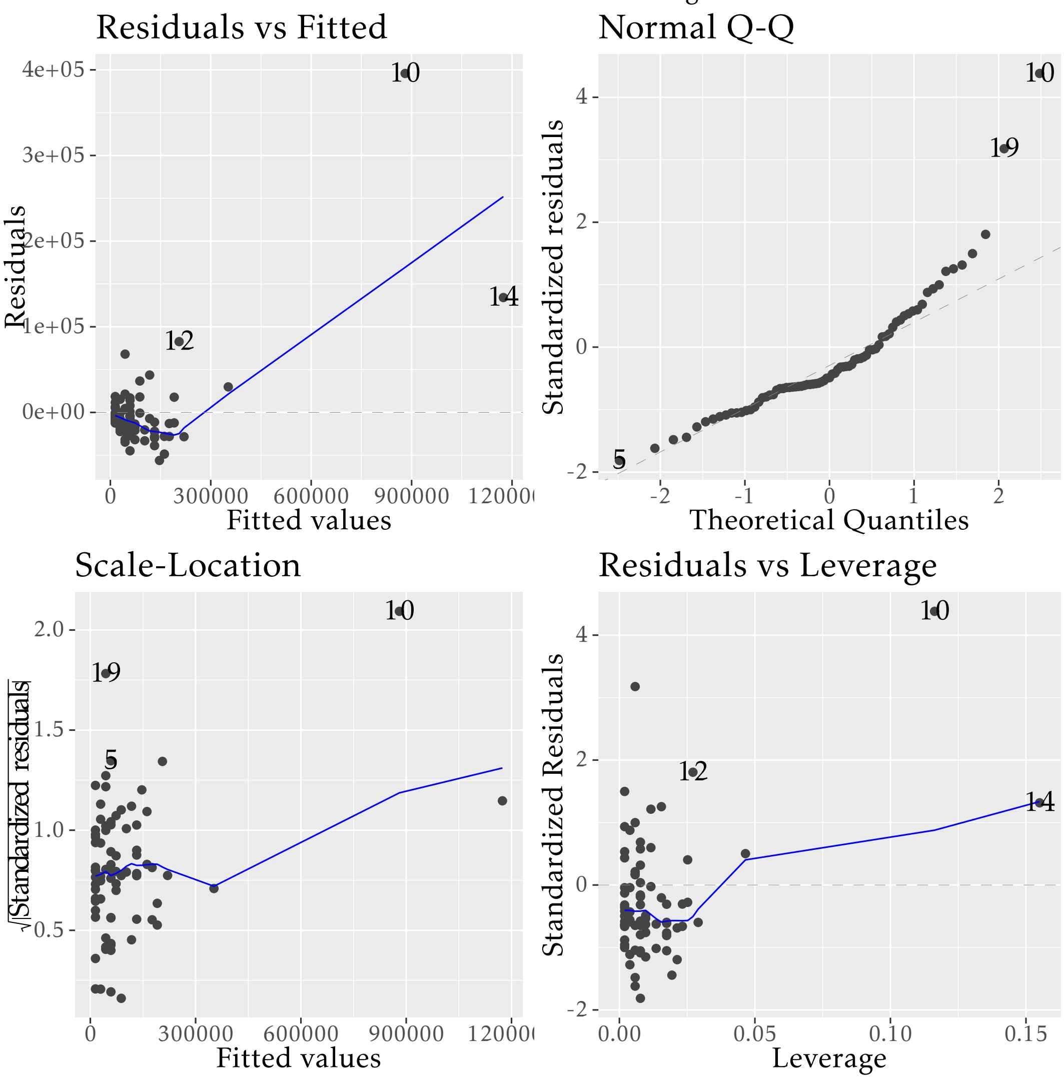
Residuals vs Fitted
4e+05
3e+05
2e+05
1e+05
0e+00
Residuals
0
300000
600000
900000
Fitted values
10
14
12
Normal Q-Q
Standardized residuals
4
2
0
-2
-1
1
Theoretical Quantiles
19
5
Scale-Location
2.0
1.5
1.0
0.5
√|Standardized residuals|
Residuals vs Leverage
Standardized Residuals
0.00
0.05
0.10
0.15
Leverage

Figure 9:

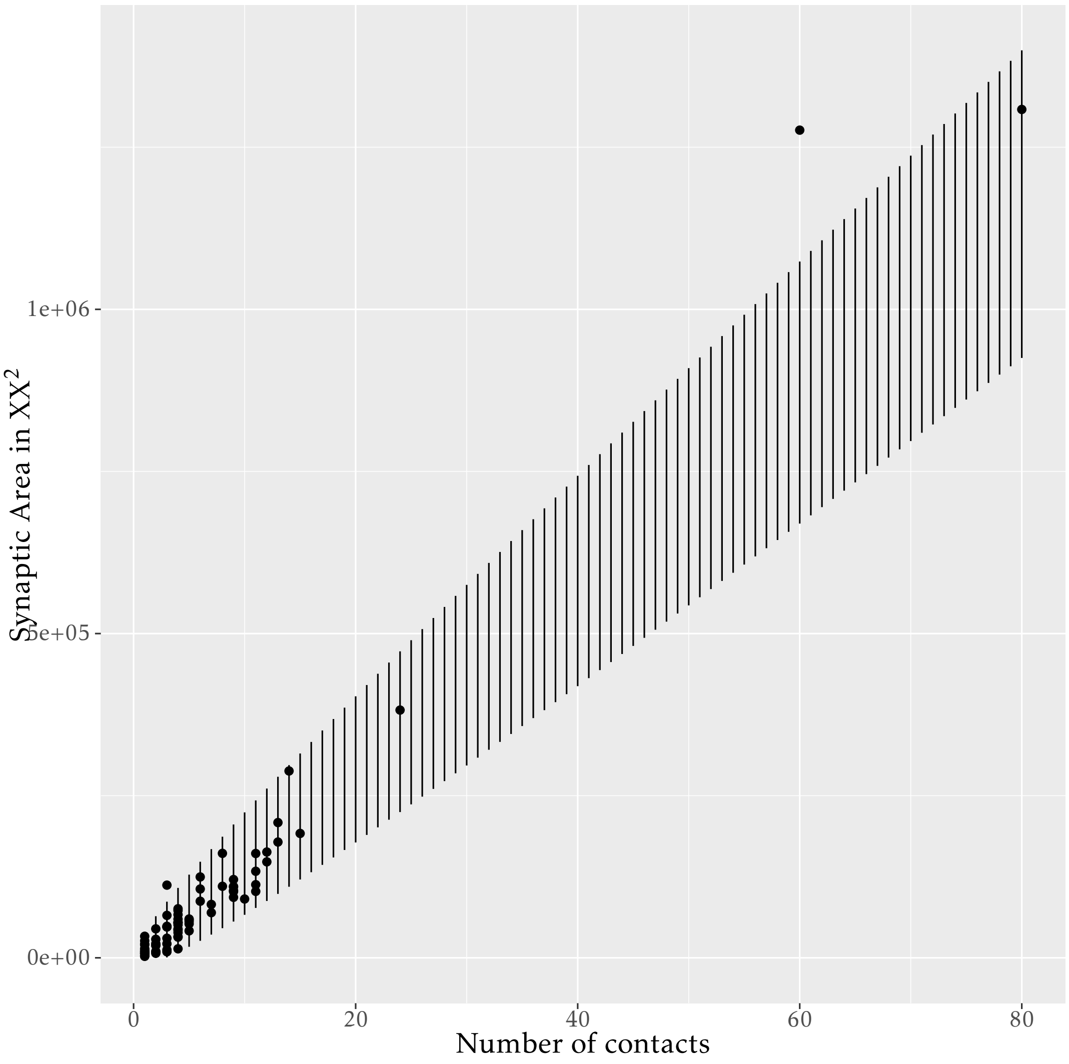
1e+06
5e+05
0e+00
Synaptic Area in XX^2
0
20
40
60
80
Number of contacts

Figure 10:

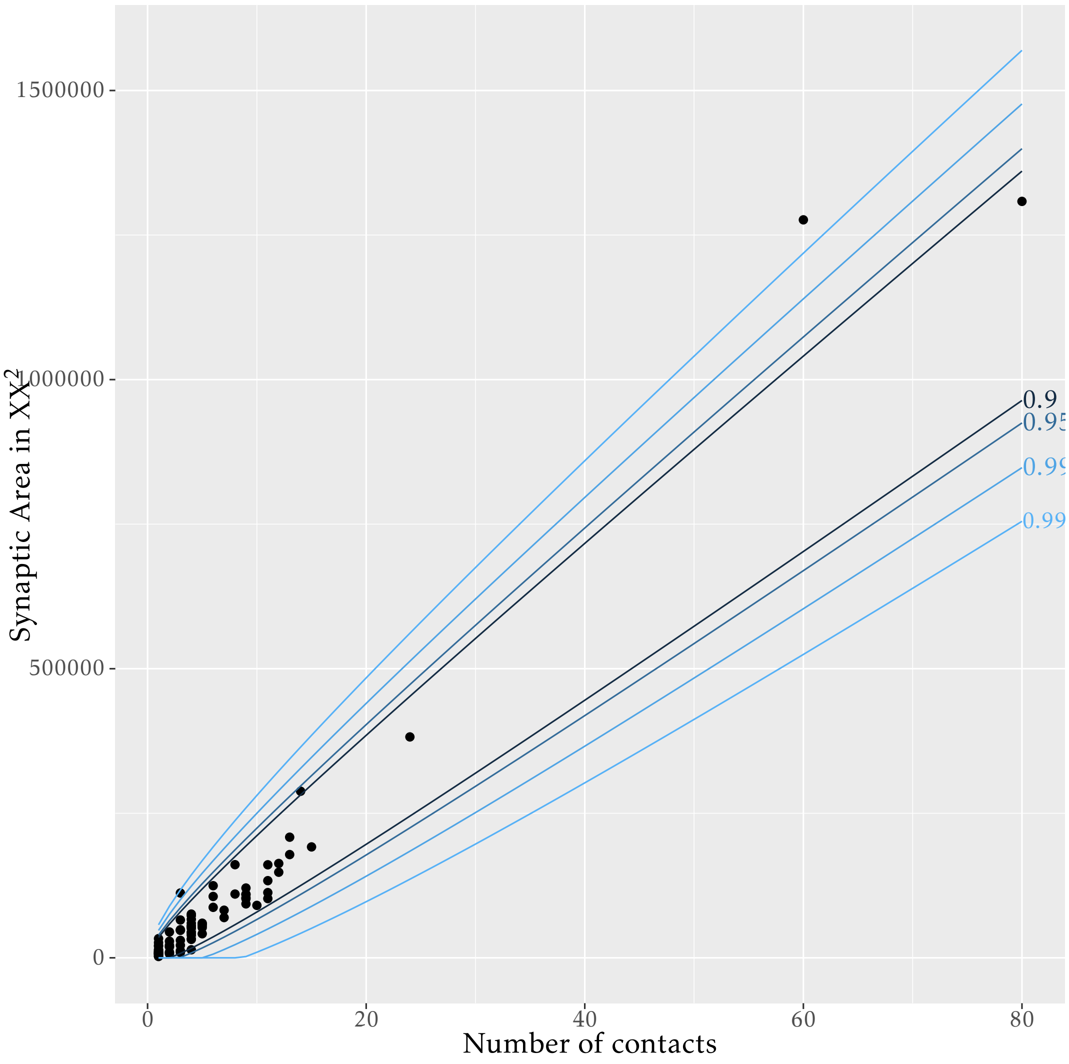
1500000
1000000
500000
0
0
20
40
60
80
Number of contacts
Synaptic Area in XX^2
0.9
0.95
0.99
0.99

Figure 11:

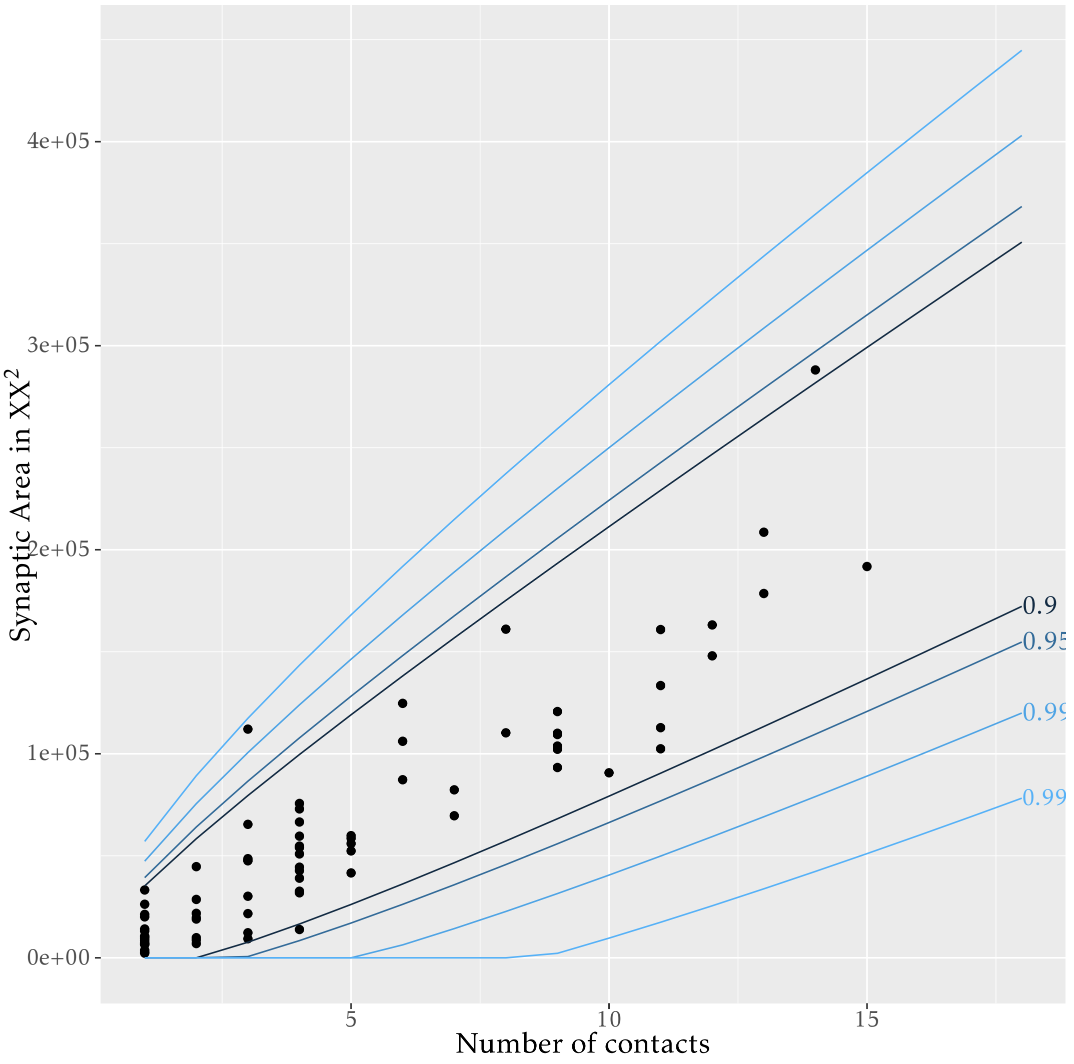
4e+05
3e+05
2e+05
1e+05
0e+00
Synaptic Area in XX^2
5
10
15
Number of contacts
0.9
0.95
0.99
0.99

6.2 Graphs from Gamma model

Figure 12:

Histogram of counts of connections per pair of neurons

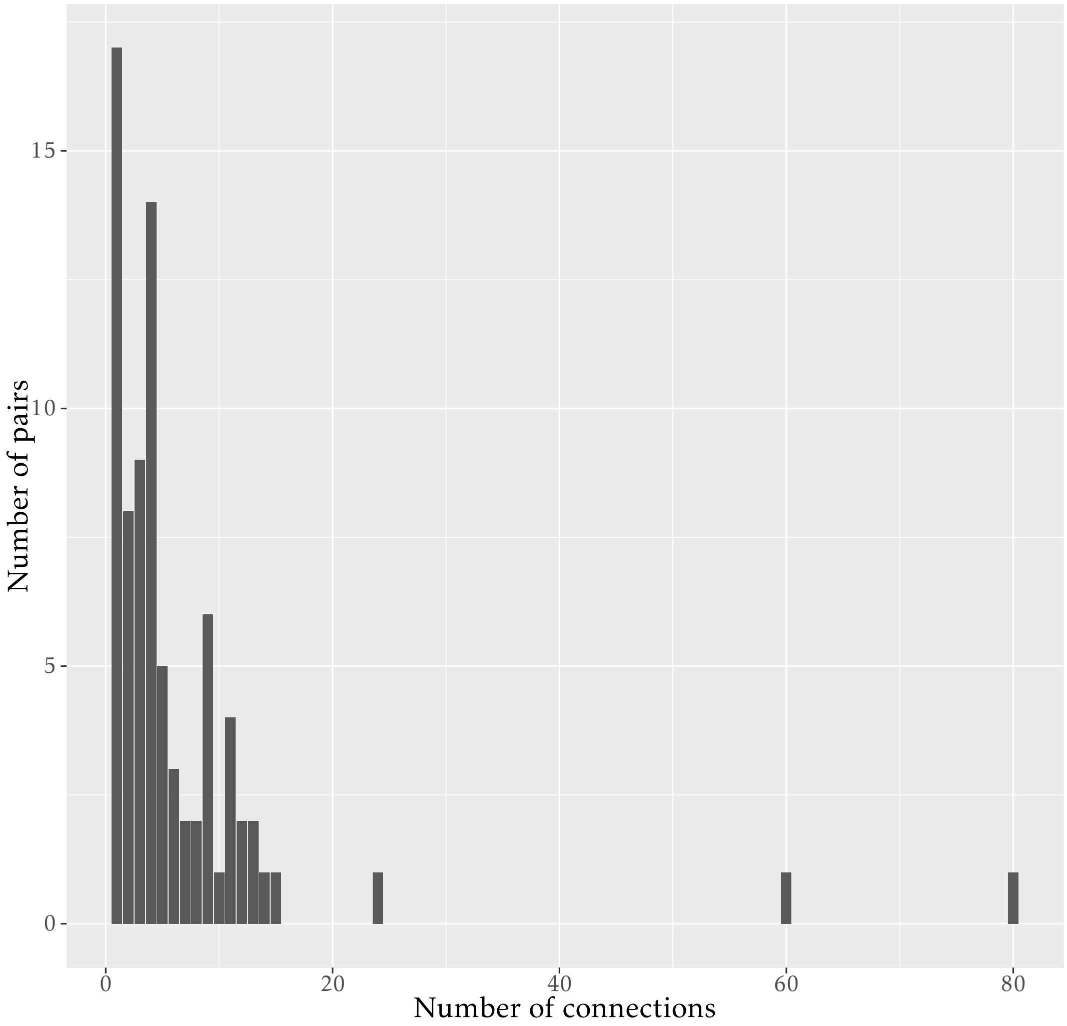

Figure 13:

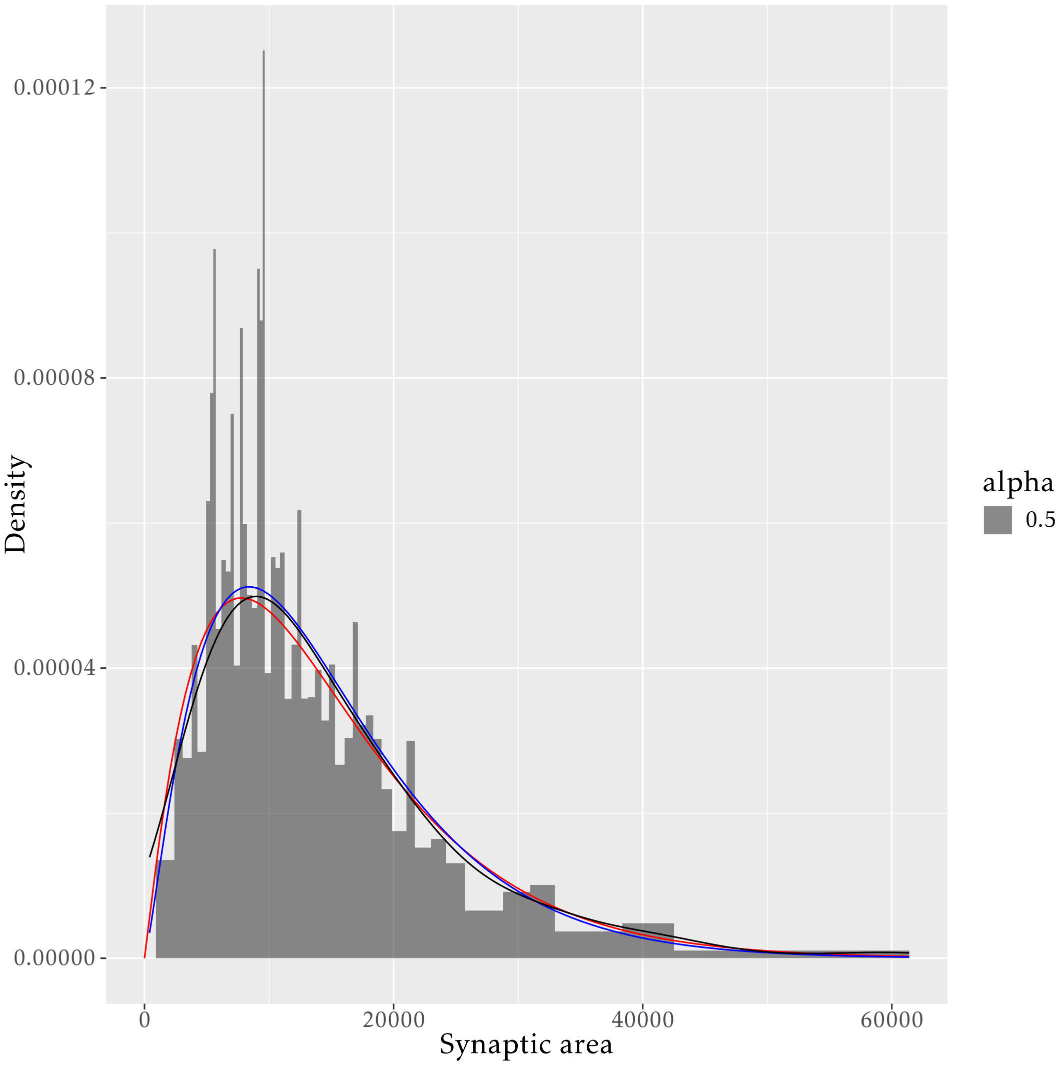
0.00012
0.00008
0.00004
0.00000
Density
0
20000
40000
60000
Synaptic area
alpha
0.5

Figure 14:

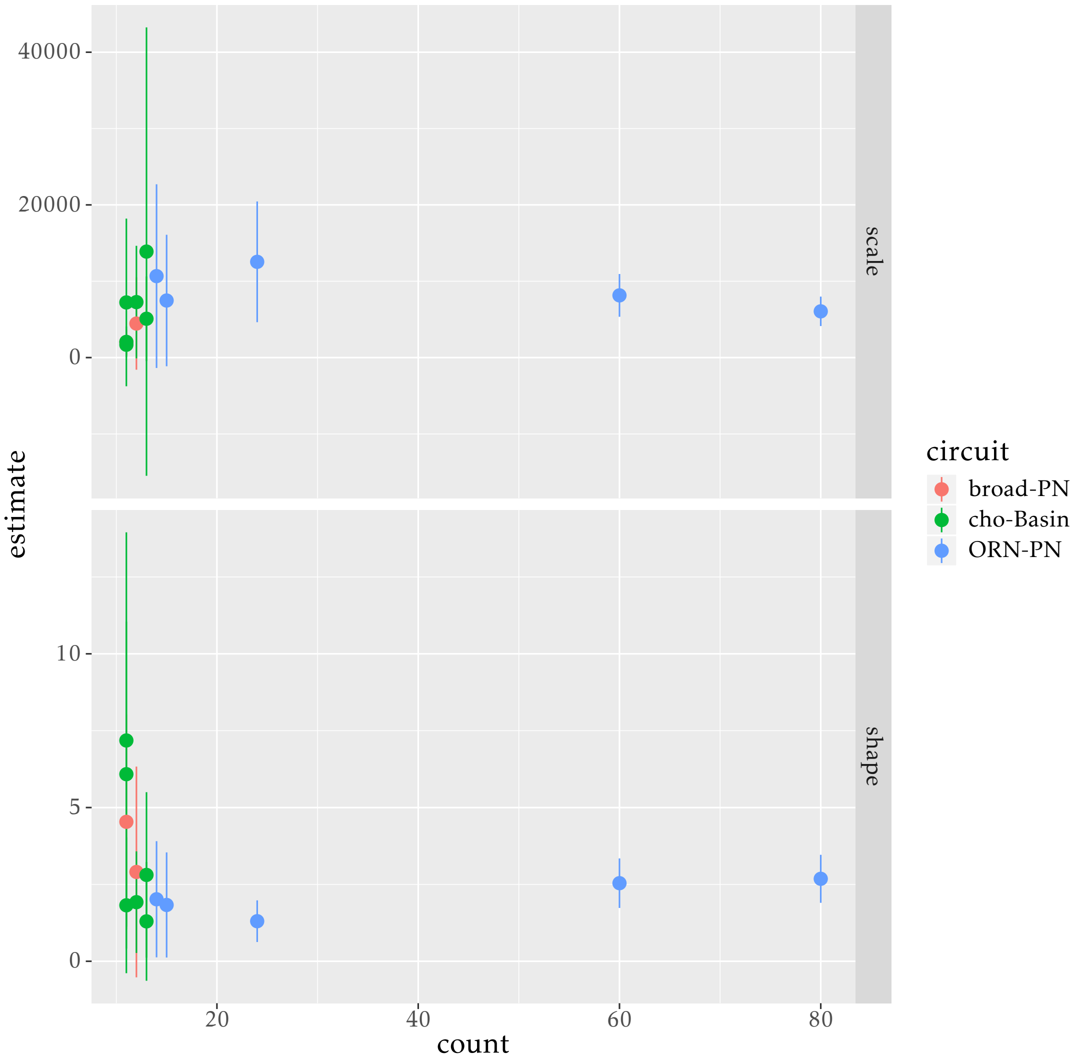
40000
20000
0
10
5
0
20
40
60
80
count
estimate
scale
shape
circuit
broad-PN
cho-Basin
ORN-PN

Figure 15:

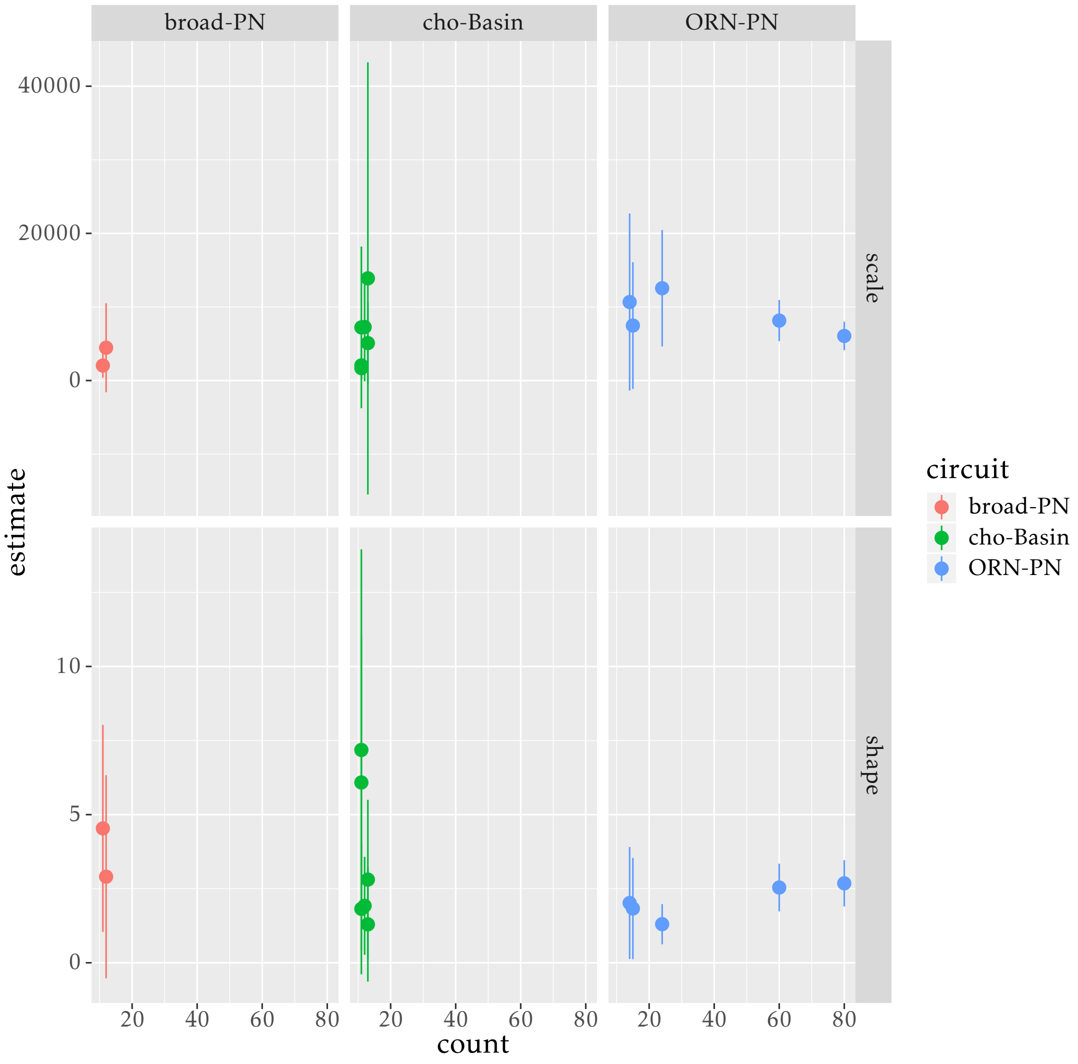
broad-PN
cho-Basin
ORN-PN
scale
shape
estimate
count
40000
20000
0
10
5
20
40
60
80
circuit
broad-PN
cho-Basin
ORN-PN

Figure 16:

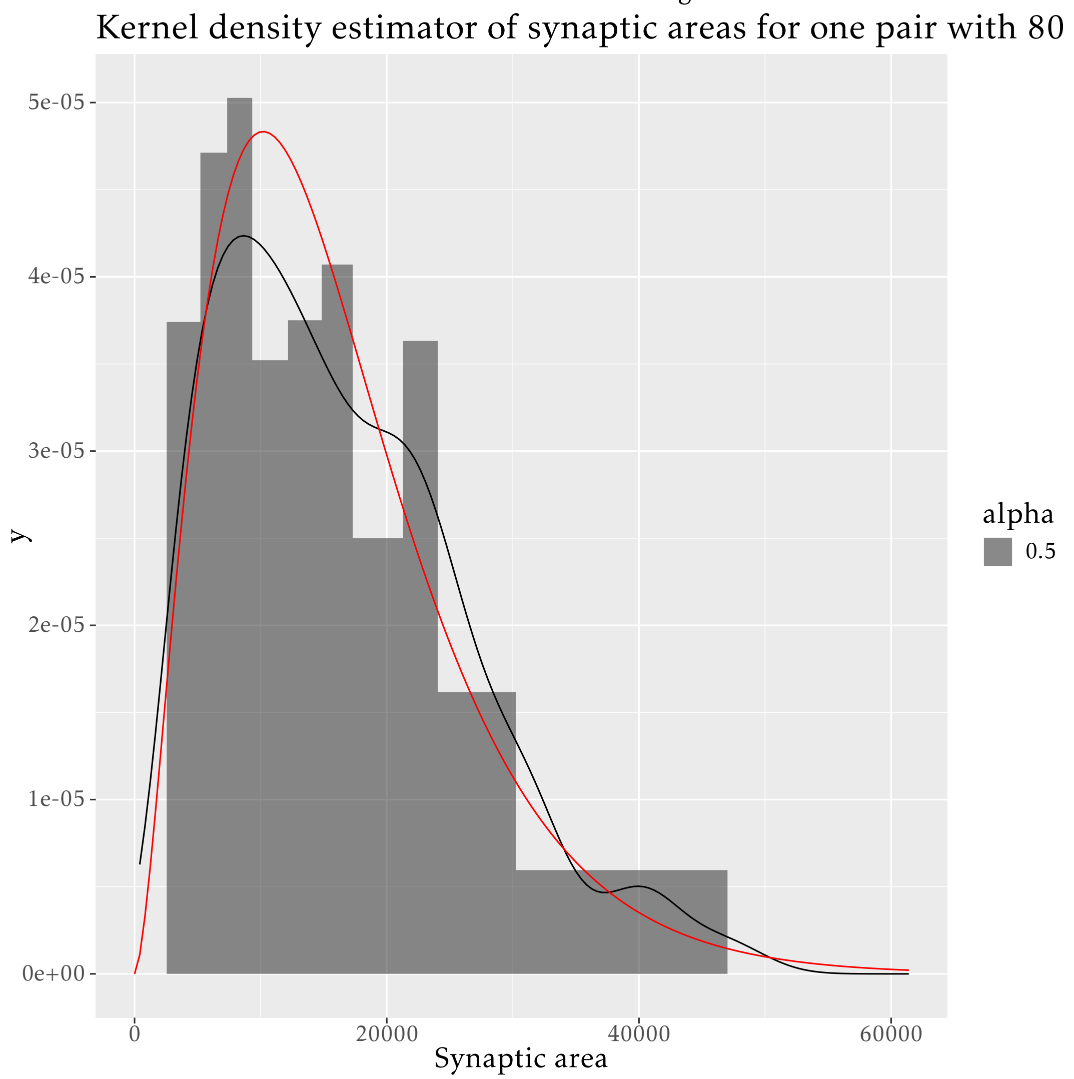

Kernel density estimator of synaptic areas for one pair with 80
5e-05
4e-05
3e-05
2e-05
1e-05
0e+00
y
0
20000
40000
60000
Synaptic area
alpha
0.5

Figure 17:

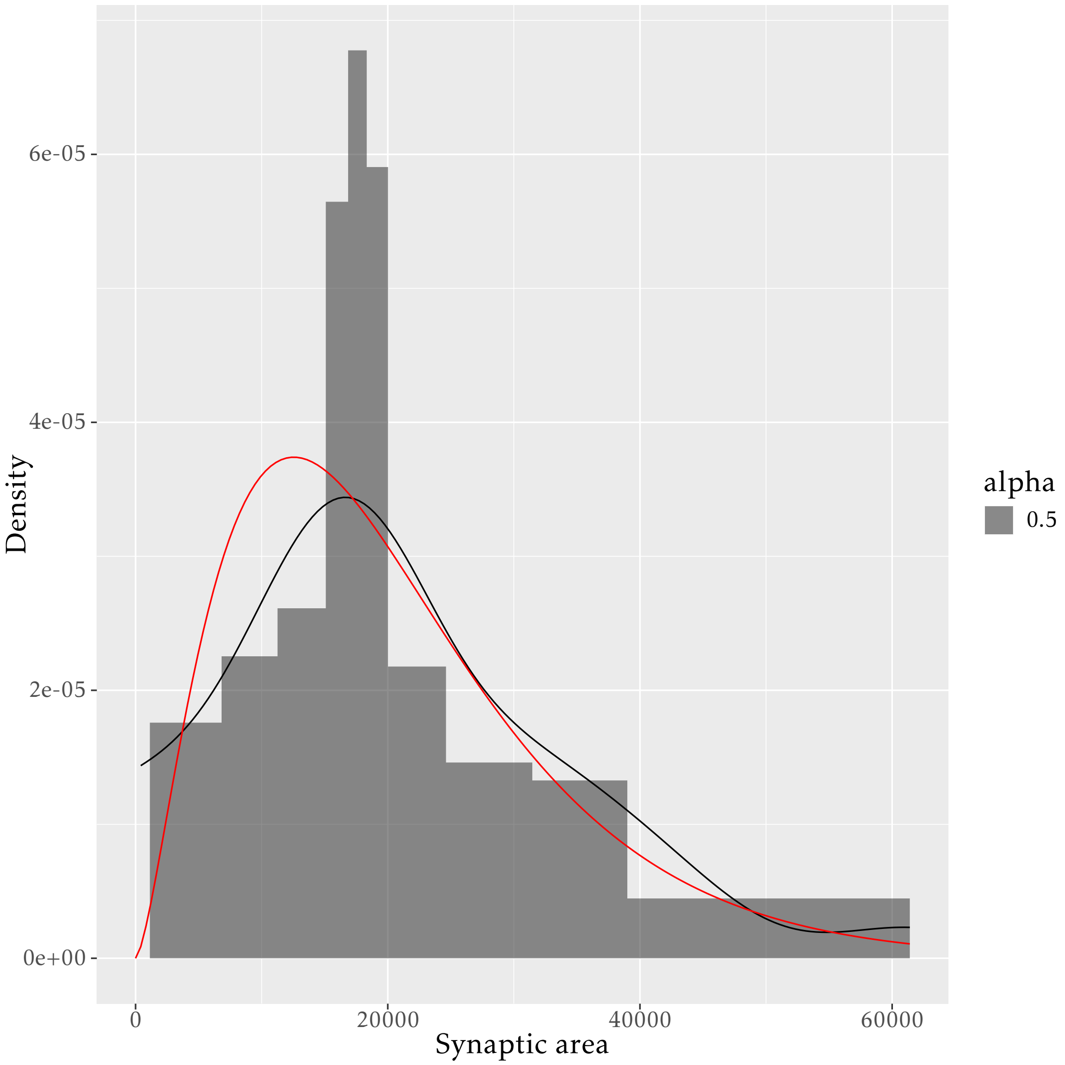
6e-05
4e-05
2e-05
0e+00
Density
0
20000
40000
60000
Synaptic area
alpha
0.5

Figure 18:

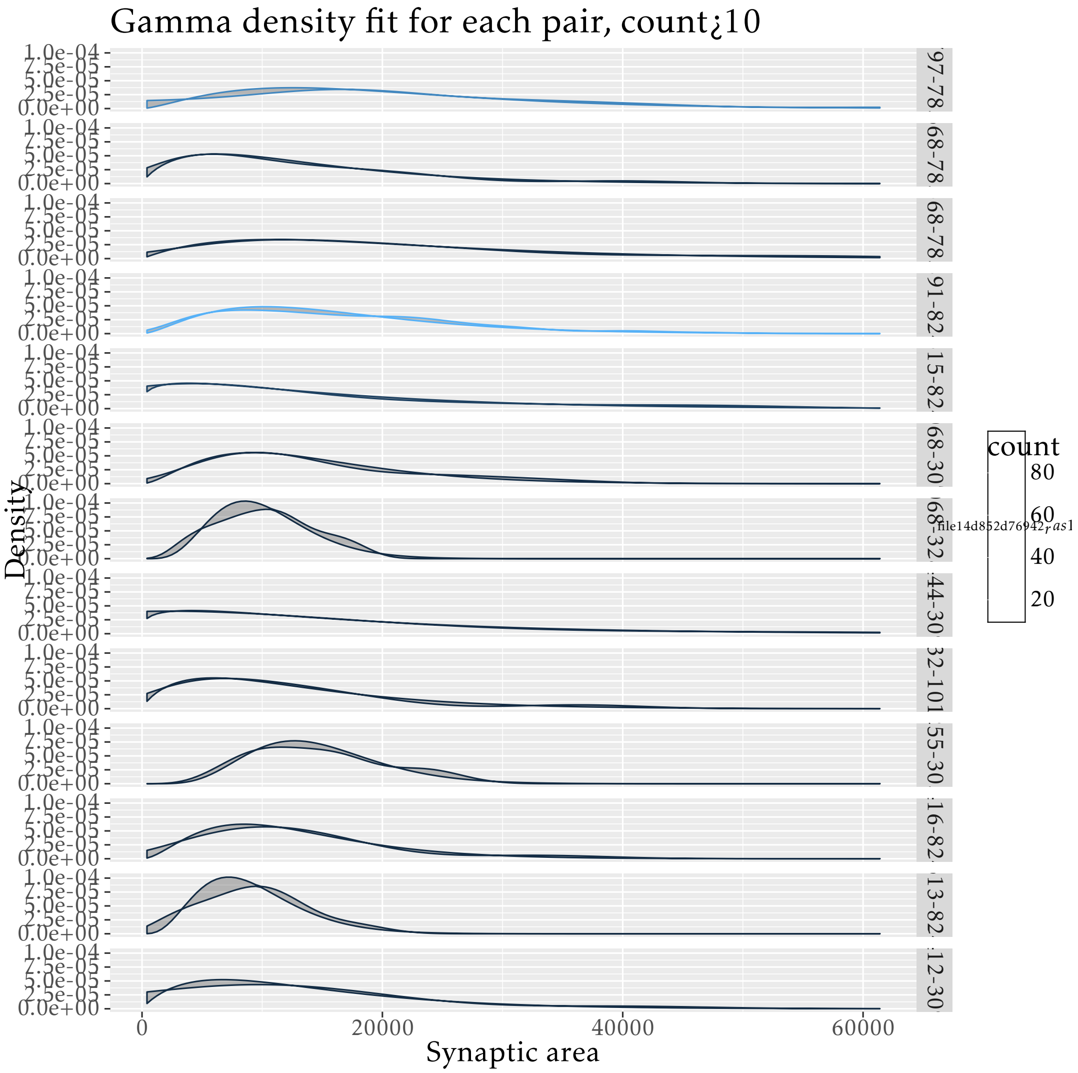
Gamma density fit for each pair, count¿10
Density
1.0e-04
7.5e-05
5.0e-05
2.5e-05
0.0e+00
97-78
68-78
68-78
91-82
15-82
68-30
68-32
44-30
32-101
55-30
16-82
13-82
12-30
0
20000
40000
60000
Synaptic area
count
80
60
40
20

Figure 19:

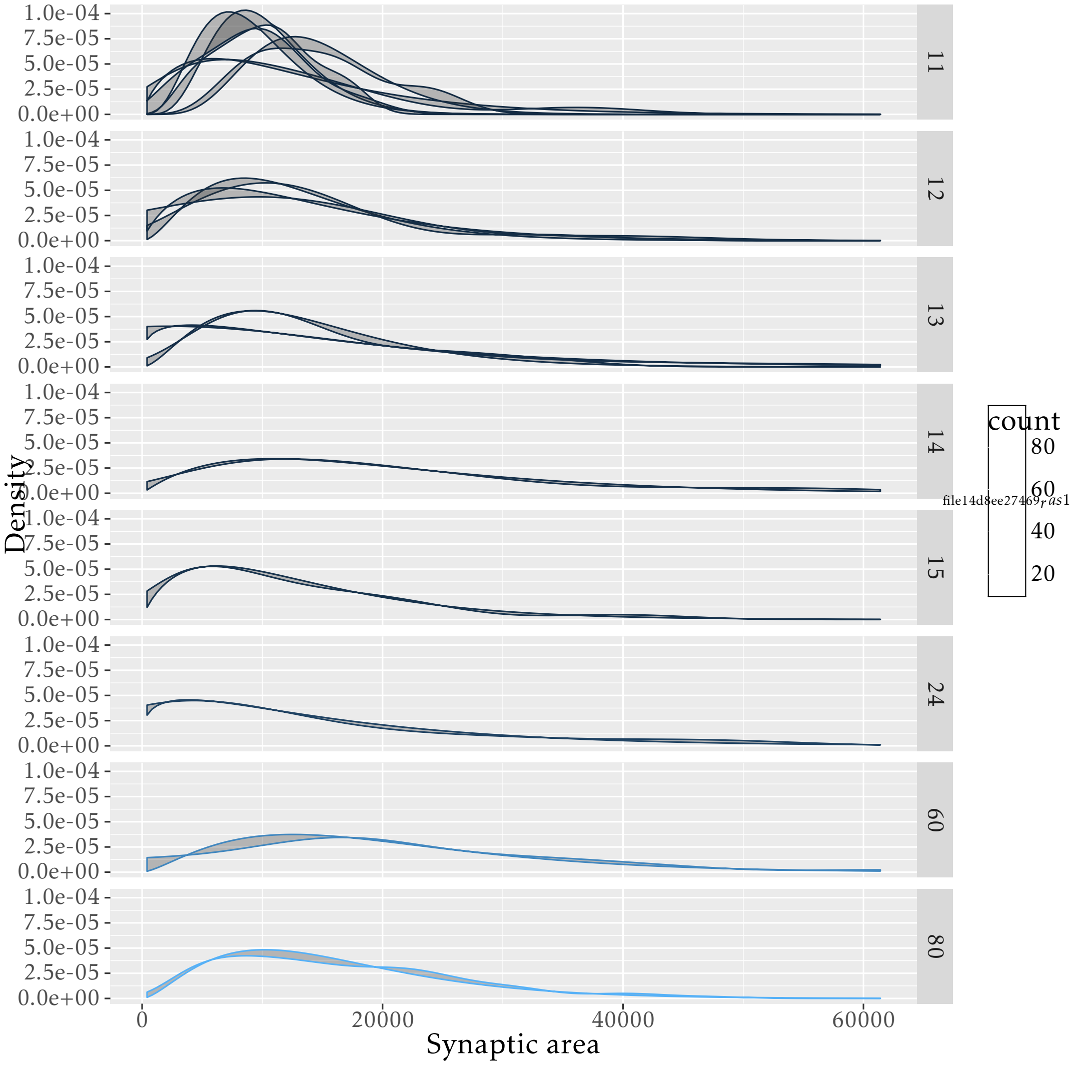
1.0e-04
7.5e-05
5.0e-05
2.5e-05
0.0e+00
11
12
13
14
15
24
60
80
Density
0
20000
40000
60000
Synaptic area
count
80
60
40
20
file14d8ee27469$_r as$1

Figure 20:

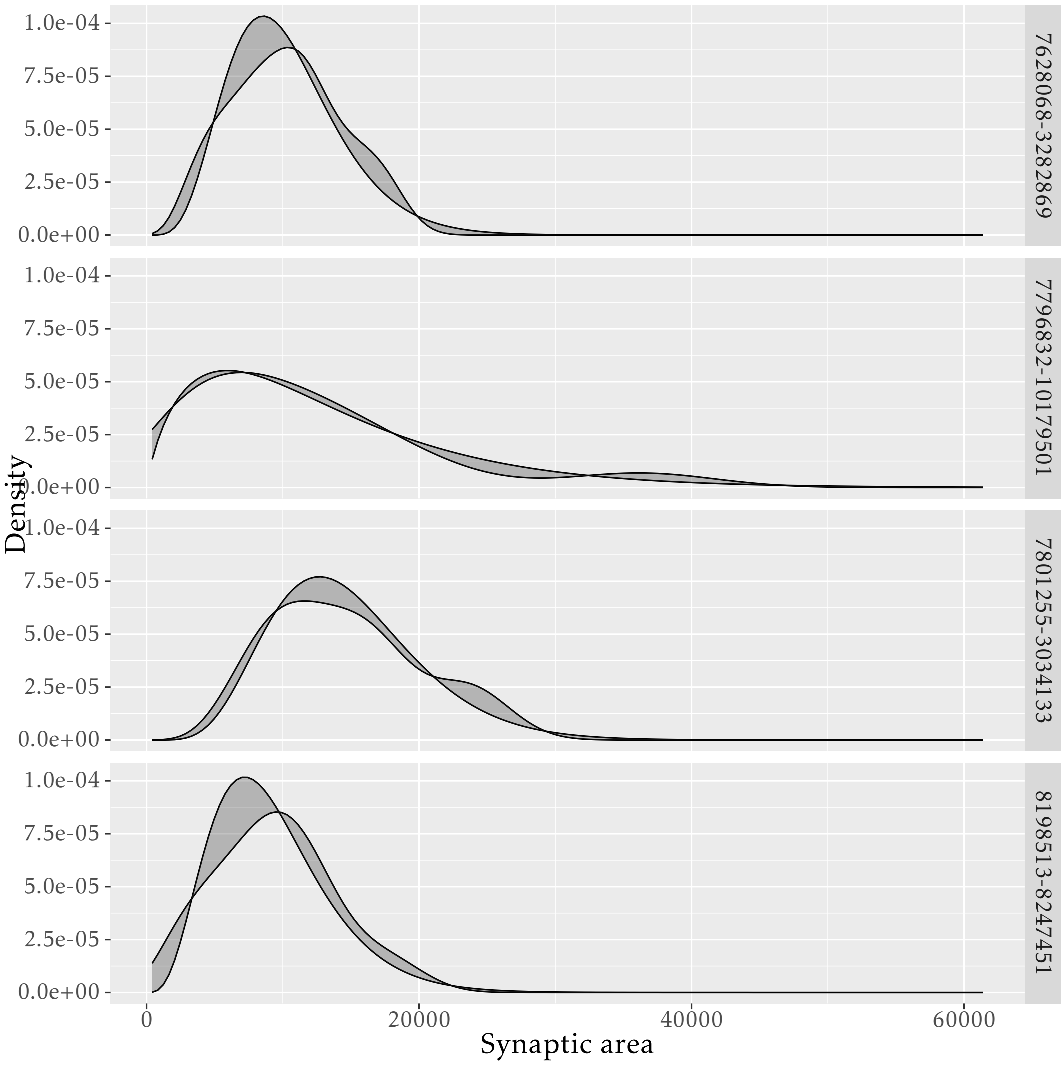
1.0e-04
7.5e-05
5.0e-05
2.5e-05
0.0e+00
7628068-3282869
7796832-10179501
7801255-3034133
8198513-8247451
Density
0
20000
40000
60000
Synaptic area

Figure 21:

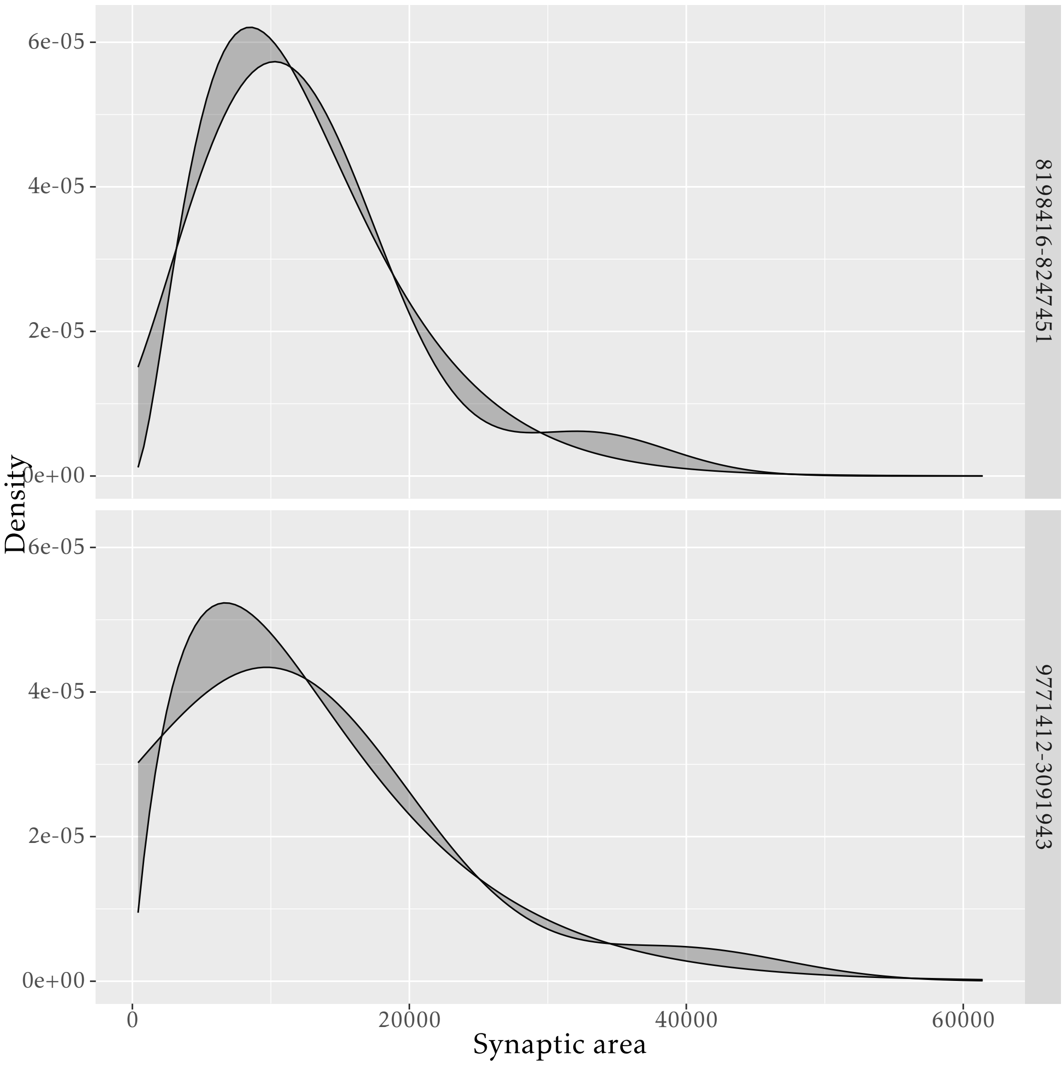
6e-05
4e-05
2e-05
0e+00
8198416-8247451
9771412-3091943
Density
0
20000
40000
60000
Synaptic area

Figure 22:

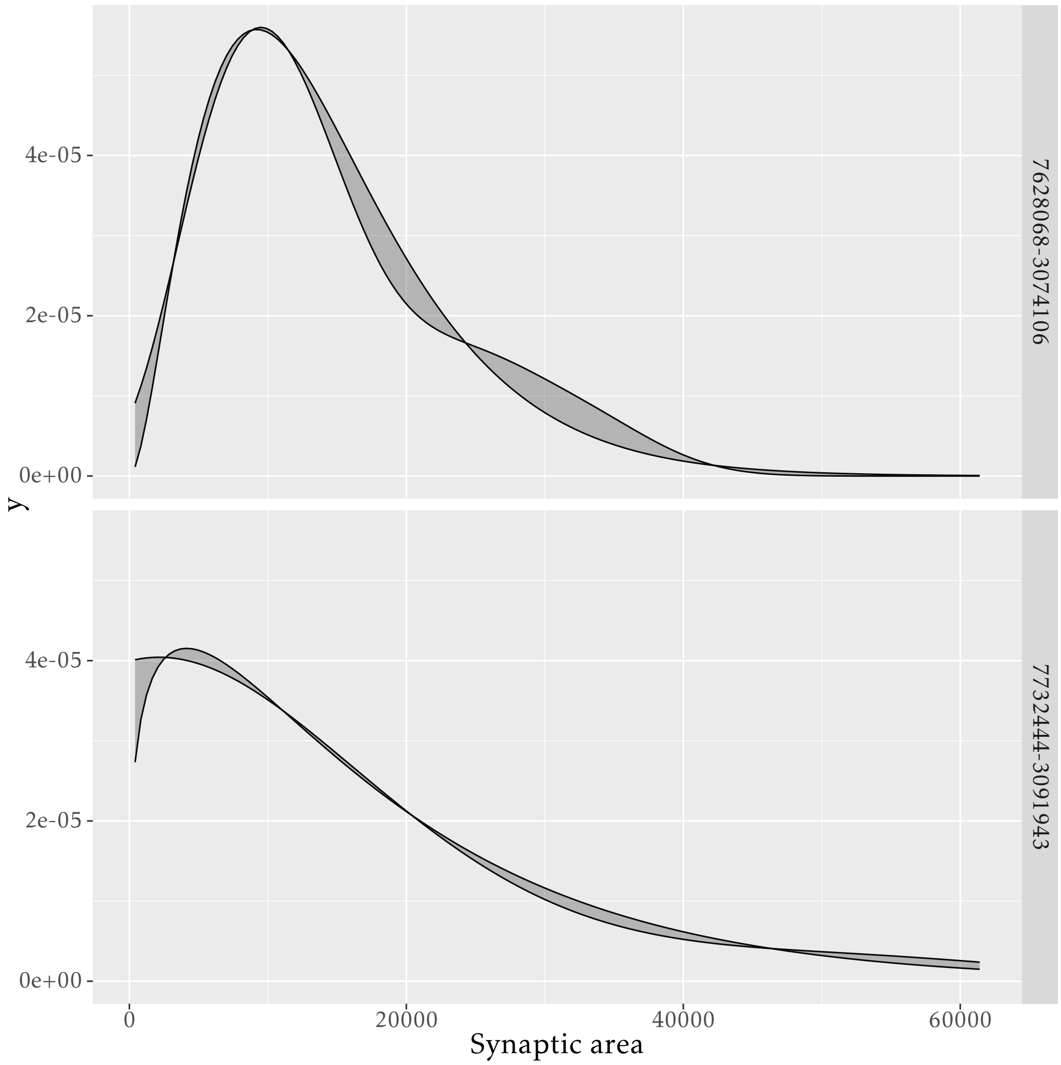
4e-05
2e-05
0e+00
y
7628068-3074106
7732444-3091943
0
20000
40000
60000
Synaptic area

Figure 23:

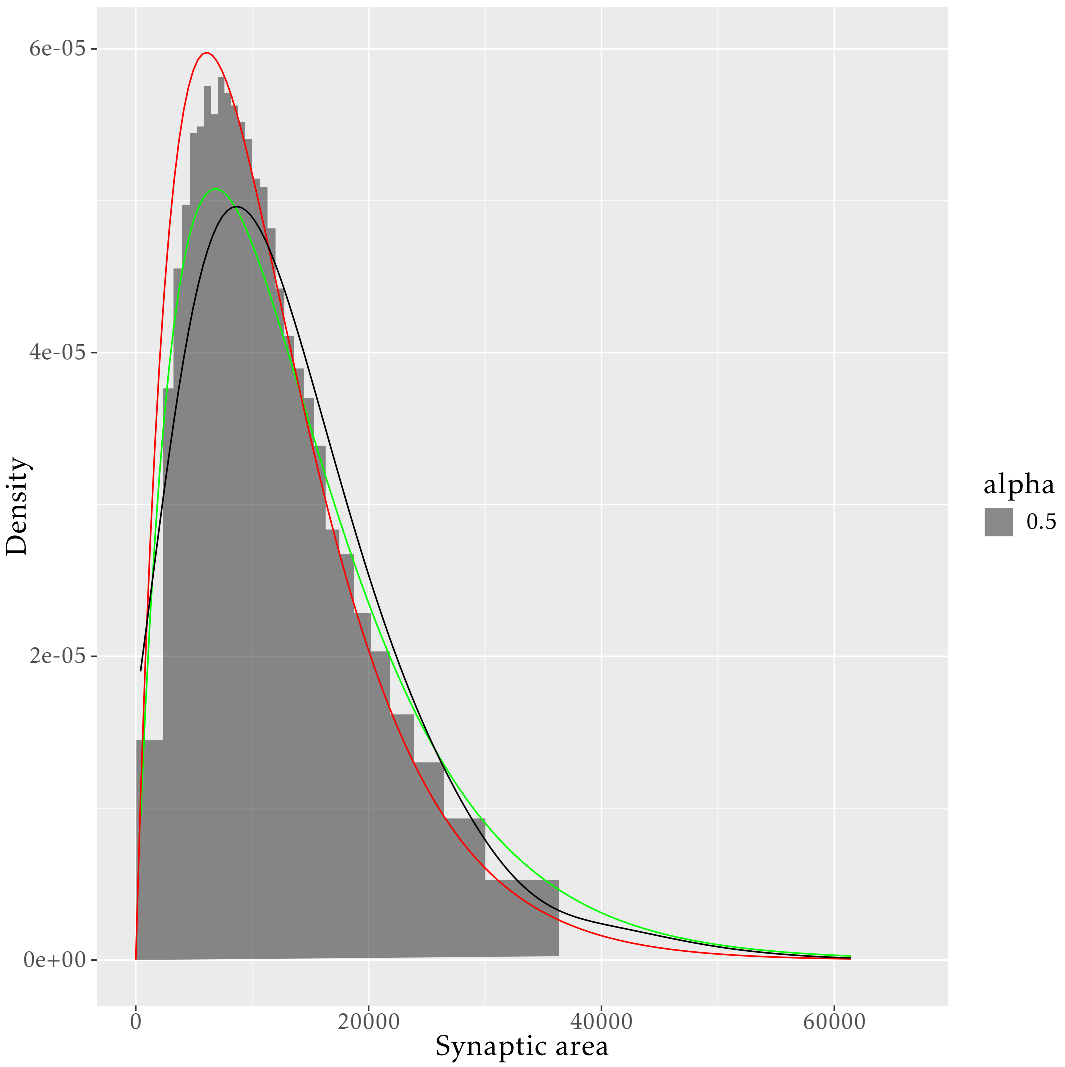

6e-05
4e-05
2e-05
0e+00
Density
0
20000
40000
60000
Synaptic area
alpha
0.5

Figure 24:

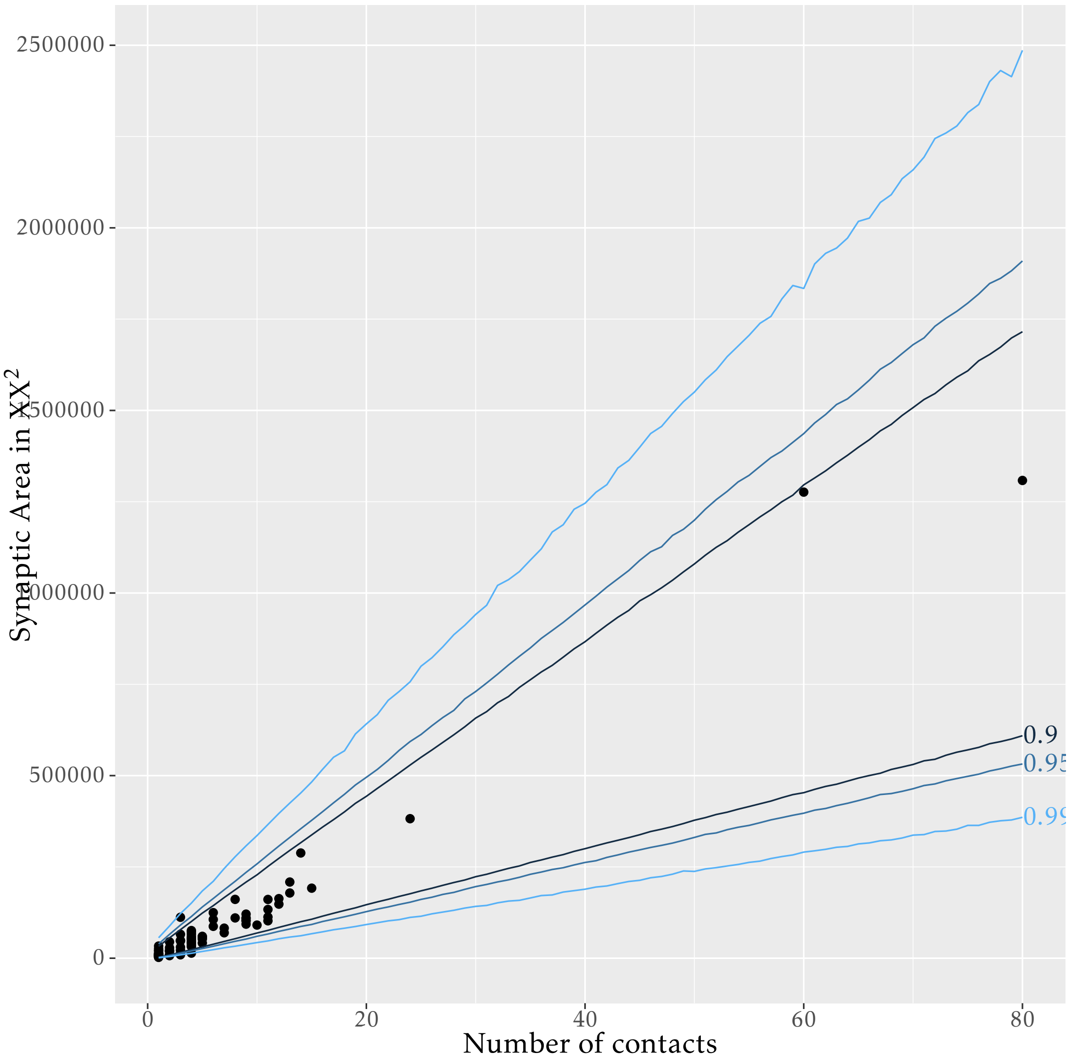
2500000
2000000
1500000
1000000
500000
0
Synaptic Area in XX^2
0.9
0.95
0.99
0
20
40
60
80
Number of contacts

Figure 25:

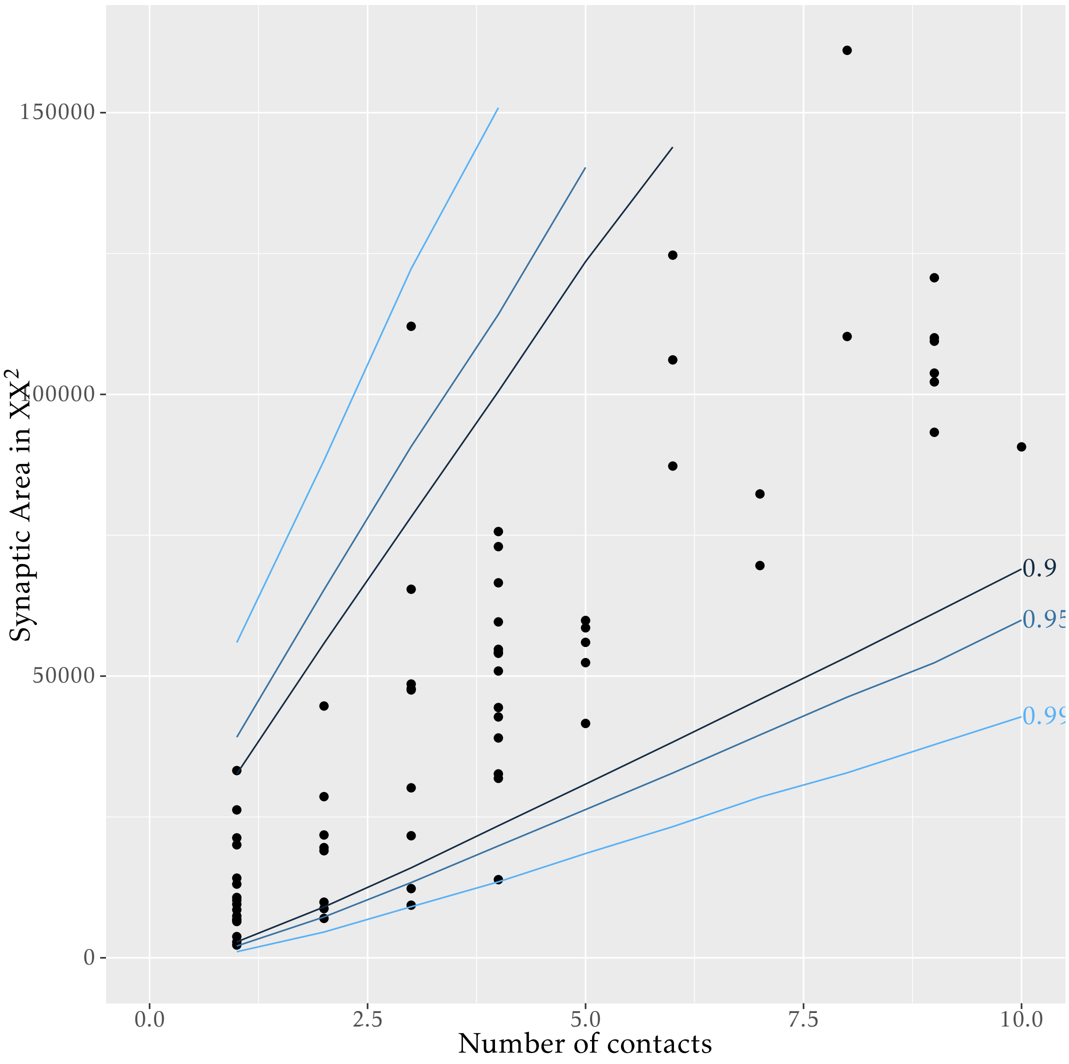
150000
100000
50000
0
0.0
2.5
5.0
7.5
10.0
Number of contacts
Synaptic Area in XX^2
0.9
0.95
0.99

Figure 26:

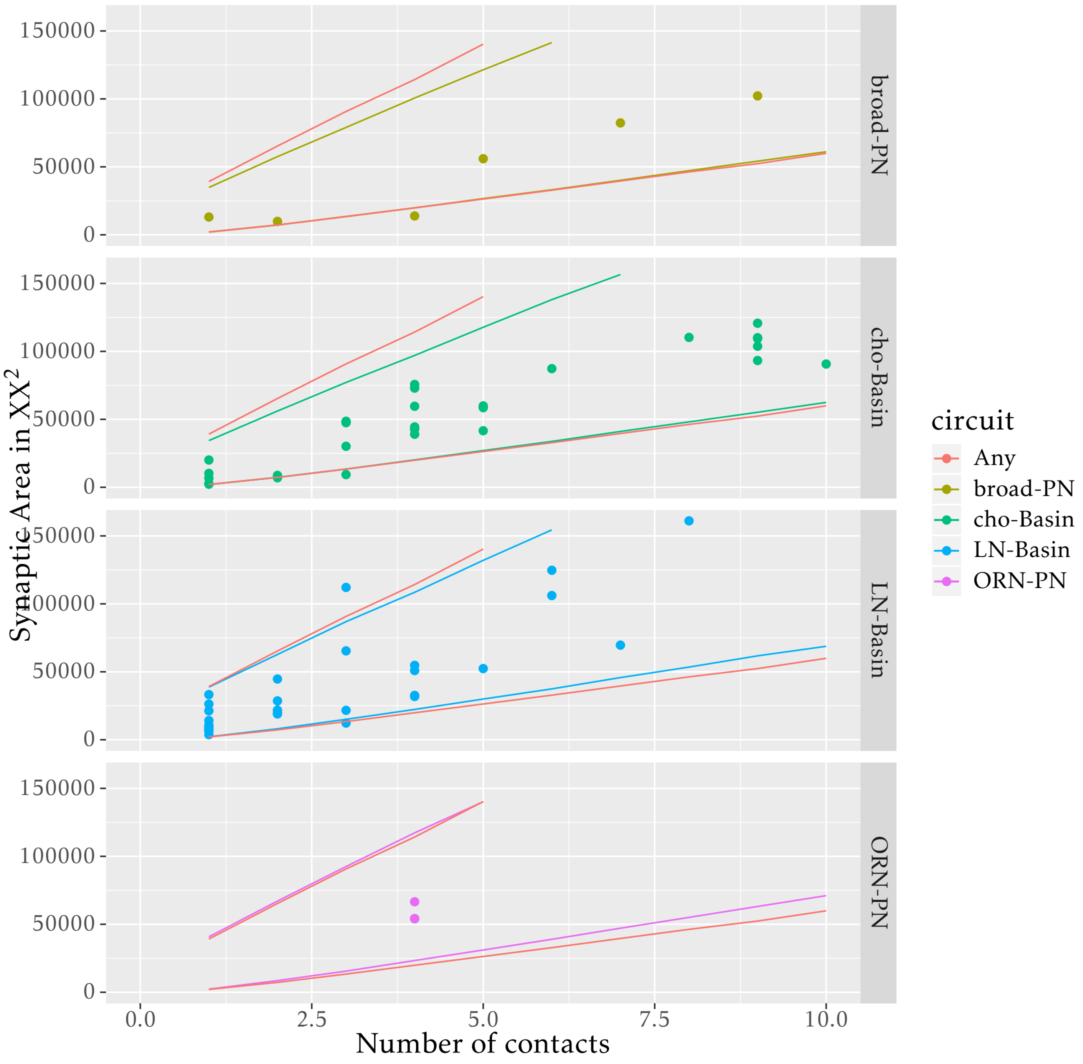

broad-PN
cho-Basin
LN-Basin
ORN-PN
150000
100000
50000
0
0.0
2.5
5.0
7.5
10.0
Number of contacts
Synaptic Area in XX²
circuit
Any
broad-PN
cho-Basin
LN-Basin
ORN-PN

6.3 Graphs from Lognormal model

Figure 27:

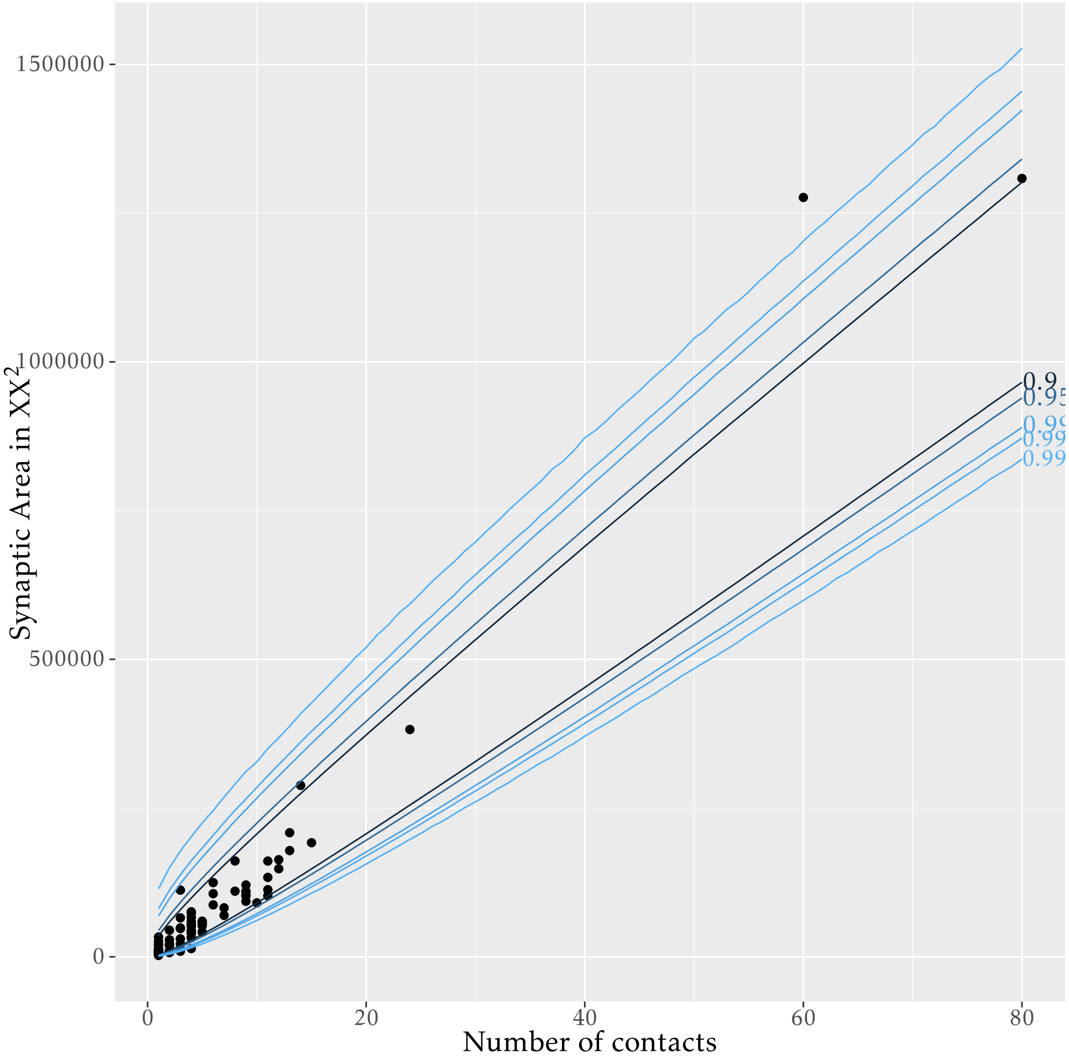

Figure 28:

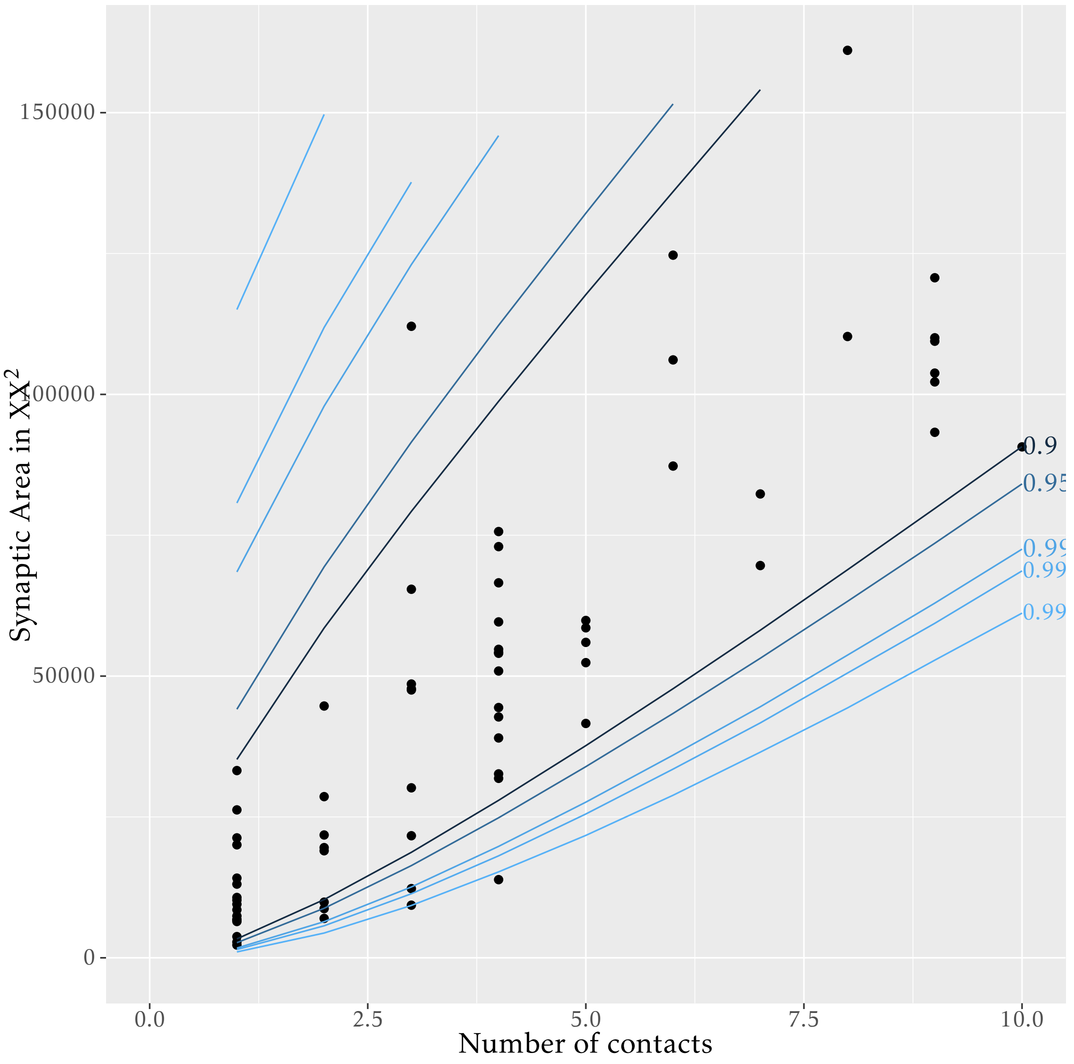

150000
100000
50000
0
0.0
2.5
5.0
7.5
10.0
Synaptic Area in XX^2
Number of contacts
0.9
0.95
0.99
0.99
0.99
