# Supplementary material for: Synaptic counts approximate synaptic contact area in Drosophila
Source: PLoS One. 2022 Apr 4;17(4):e0266064. doi: 10.1371/journal.pone.0266064 (PMC8979427; doi:10.1371/journal.pone.0266064)
Supplement: Attachment — Submitted filename: Response to Reviewers.pdf [file pone.0266064.s003.pdf]

Dr. Albert Cardona, Programme Leader
MRC Laboratory of Molecular Biology (LMB)
and Reader (Associate Professor), University of Cambridge
Cambridge Biomedical Campus
Francis Crick Ave
Cambridge CB2 0QH
acardona@mrc-lmb.cam.ac.uk

March 10, 2022

Dear editors,

We thank the reviewers for their valuable comments on our manuscript and have made edits to address their concerns. The review comments are below in blue.

> Barnes et al demonstrate that that excitatory and inhibitory inputs onto Olfactory projections neurons (PNs) and first-order mechano/nociceptive neurons (Basins) exhibit a very simple relationship between the number of contacts and the total surface area of the synaptic surface area. The data is rigorously analysed, well presented and the conclusions drawn from this work are clearly stated. In their conclusions, the authors argue that simply measuring the number of synaptic contacts between neuron A and B could be used as a surrogate marker of synaptic strength. If true, this would greatly simplify some of the challenges associated with connectome analysis.
> I was surprised that no mention was made of Peters rule – a term that was applied by others to the work of Peters and Feldman (J Neurocytol. 1976 Feb; 5(1):63-84) – that close apposition of axons to dendrites can be used to predict the number of synapses. This simple rule is still widely debated and not at all proven. The data presented by Barnes et al builds on Peters rule as they show that the number of contacts would correlate well with synaptic area.

Thank you for highlighting the relationship between our work and Peters' Rule. We agree that in cases where Peter's Rule holds, the importance of our findings would be bolstered as imaging modalities could be even further optimised for rapid acquisition of neurite without even needing to resolve synaptic puncta. We have added language to clarify that, due to evidence suggesting that it does not hold across some circuits [1, 2], synapses should still be counted rather than inferred.

> However, my main concern with this manuscript is that there is a lack of direct evidence cited in the current manuscript to support the view that synaptic area is a measure of synaptic strength. Barnes et al rely on three very important research papers to support this key argument. Unfortunately, I do not see how any of these papers support this idea. The seminal work of Castillo & Katz in the 1950's is somehow used to make the claim that synaptic strength correlates with release probability. I cannot see how this claim can be made from the elegant studies on the quantal nature of the miniature end plate potential by Castillo & Katz.

We are grateful for pointing out the unclear wording around the Castillo and Katz [3] citation, which we have now resolved.

We have generally improved the clarity of the reasoning relating synaptic area to synaptic strength. Firstly, this has been shown directly in mammalian neocortex by Holler et al. [4].

The authors then cite Branco et al (2010) to claim that vesicle release probability correlates with the number of docked vesicles. However, the paper by Branco et al (2010) reached entirely the opposite conclusion about synaptic area and release probability. This paper clearly states that although there is a positive correlation between release probability and the number of docked vesicles the synaptic area does not correlate with the release probability.

Branco, Marra, and Staras [5] indeed showed that the total number of vesicles near the synaptic terminal (their fig. 5c), which can be estimated from the volume of the terminal, does not correlated with release probability. Branco, Marra, and Staras [5] also showed that the number of docked vesicles correlates linearly with release probability (their fig. 5b): this is what correlates with the area of the presynaptic zone and we felt was more relevant to our study, which we have now clarified in the text.

The final paper (Ikeda & Bekkers, 2009) does not contain any anatomical data concerning docked vesicles and uses a purely functional approach based upon blocking transmitter recycling to estimate the reserve pool of vesicles and I do not see the relevance of this paper to the arguments made by Barnes et al.

We have clarified the relevance of this paper, especially as it relates to the work of Branco et al. above. A significant but highly variable proportion of the total number of vesicles near the synaptic terminal are in the reserve pool, which Ikeda and Bekkers [6] showed were only mobilised under prolonged stimulation.

Therefore, I feel that this aspect of the conclusion needs a lot more work.

The conclusion has now been reworked to better represent our chain of reasoning in relating synaptic area to strength.

There are many other studies that have attempted to address the question of whether synaptic area correlates with synaptic strength, but these studies were overlooked in the current manuscript. In particular, I am reminded of the work of Farrant, Cull-Candy & Nusser (1997). This study combined whole-cell recording with quantitative immunolabelling at EM resolution to conclude that variation in receptor number at the synapse largely explains variability in mIPSC amplitude. Importantly for Barnes et al, receptor density appears uniform and so surface area could be used to predict receptor number.

We appreciate highlighting this important work and we have now incorporated it into our discussion.

Unfortunately, receptor density may not be uniform at all synapse types and I am not aware this parameter is known for Drosophila – it certainly is not reported in the current manuscript. In the absence of this data or any functional data on the synaptic strength recorded at the excitatory and inhibitory synapses onto PN and Basin neurons of Drosophila I am concerned that the importance of this work is over-stated. However, the analysis performed on the data-sets in this study are impressive and I am sure this work will be of

great value to many involved in connectome research.

We agree that obtaining further functional data and area measurements across more cell types will be important going forward. We note that a number of groups are already using synaptic counts as a proxy for edge weights in *Drosophila* in circuits containing a variety of cell types. We have edited the discussion to better state that our data supports the continued use of this assumption, but that more measurements, both functional and anatomical, will further reinforce our findings and simultaneously identify circuits where such an approach does not apply. Examples of exceptions would include very low synapse count edges, such as in *C. elegans* and in cerebellar mossy fibre - granule cell synapses.

We hope that these improvements have addressed the reviewers' concerns and would like to again state our appreciation for your considered and valuable comments.

Sincerely,

Albert Cardona

References

[1] Narayanan Kasthuri et al. "Saturated Reconstruction of a Volume of Neocortex". In: *Cell* 162.3 (July 2015), pp. 648–661. ISSN: 10974172. DOI: 10.1016/j.cell.2015.06.054.

[2] Casey M Schneider-Mizell et al. "Quantitative neuroanatomy for connectomics in Drosophila". In: *eLife* 5.MARCH2016 (2016). ISSN: 2050084X. DOI: 10.7554/eLife.12059. URL: https://www.ncbi.nlm.nih.gov/pmc/articles/PMC4811773/pdf/elife-12059.pdf.

[3] J. del Castillo and B. Katz. "Quantal components of the end-plate potential". In: *The Journal of Physiology* 124.3 (June 1954), pp. 560–573. ISSN: 14697793. DOI: 10.1113/jphysiol.1954.sp005129. URL: https://www.ncbi.nlm.nih.gov/pmc/articles/PMC1366292/.

[4] Simone Holler et al. "Structure and function of a neocortical synapse". In: *Nature* 591.7848 (Dec. 2021), pp. 111–116. ISSN: 14764687. DOI: 10.1038/s41586-020-03134-2. URL: https://doi.org/10.3929/ethz-b-000394586.

[5] Tiago Branco, Vincenzo Marra, and Kevin Staras. "Examining size-strength relationships at hippocampal synapses using an ultrastructural measurement of synaptic release probability". In: *Journal of Structural Biology* 172.2 (Nov. 2010), pp. 203–210. ISSN: 10478477. DOI: 10.1016/j.jsb.2009.10.014. URL: https://www.sciencedirect.com/science/article/pii/S1047847709003025.

[6] Kaori Ikeda and John M. Bekkers. "Counting the number of releasable synaptic vesicles in a presynaptic terminal". In: *Proceedings of the National Academy of Sciences of the United States of America* 106.8 (Feb. 2009), pp. 2945–2950. ISSN: 00278424. DOI: 10.1073/pnas.0811017106. URL: www.pnas.org/cgi/content/full/.
